# Towards contextualized complex systems approaches to scaling-up hepatitis B birth-dose vaccination in the African region: a qualitative systematic review

**DOI:** 10.3389/fpubh.2024.1389633

**Published:** 2024-10-24

**Authors:** Tasneem Solomon-Rakiep, Jill Olivier, Edina Amponsah-Dacosta

**Affiliations:** ^1^Health Policy and Systems Division, School of Public Health, Faculty of Health Sciences, University of Cape Town, Observatory, Cape Town, South Africa; ^2^Vaccines for Africa Initiative, School of Public Health, Faculty of Health Sciences, University of Cape Town, Observatory, Cape Town, South Africa

**Keywords:** Africa, birth-dose, complexity, health systems, hepatitis B, maternal and child health, vaccine

## Abstract

**Background:**

Despite the longstanding implementation of universal hepatitis B infant vaccination programs, the World Health Organization African region (WHO AFRO) maintains the highest prevalence (2.5%) of chronic hepatitis B virus (HBV) infection among children ≤5 years of age. Scaling-up hepatitis B birth-dose (HepB BD) vaccination could avert mother-to-child transmission of HBV infection and advance regional progress towards eliminating viral hepatitis.

**Objective:**

To describe whether – *and how* – complexities within the health system or intervention influence the performance of HepB BD vaccination programs in the WHO AFRO.

**Methods:**

Using a complexity perspective, we conducted a qualitative systematic review of literature published between 2009–2022. A Boolean search strategy retrieved relevant literature indexed in PubMed, EBSCOhost databases, Scopus, and Web of Science, with supplementary searches conducted to identify any missed articles. No language restrictions were applied. Data extraction, synthesis and analysis were guided by a systems-based logic model tailored to systematic reviews of complex interventions.

**Results:**

Our search yielded 672 published records. Of these, 28 (26 English, 2 French) were eligible for inclusion. Among the 12 WHO AFRO member states represented, the origin of evidence weighted highest in Nigeria (*n* = 12) and Senegal (*n* = 5). The performance of HepB BD vaccination programs across member states are influenced by underlying complexities across eight cross-cutting themes: (i) availability and interpretation of HepB BD vaccination policies, (ii) capacity of vaccine supply and cold chain systems, (iii) availability of equitable and sustainable financing, (iv) capacity and capability of health care workers (HCWs), (v) immunization monitoring systems and impaired feedback loops, (vi) influence of context vs system design on the timeliness of vaccination, (vii) maternal knowledge and socio-economic factors, and (viii) wider contextual factors (geography, climate, cultural practices).

**Conclusion:**

Countries looking to introduce, or scale-up HepB BD vaccination programs will benefit from careful consideration of components of the intervention design that are dependent on the end-user’s context and capabilities in accessing the vaccine; the adherence and interpretation of essential components of the policy; the provision of adequate support of stakeholders specifically HCWs and government ministries; and the need for innovative approaches to underlying complexities. Lessons offered by these African experiences provide pragmatic approaches to successfully implementing HepB BD vaccination programs in the region.

## 1 Introduction

Vaccination of newborns within 24 h of life with a single dose of the hepatitis B vaccine is pivotal to preventing mother-to-child-transmission (MTCT) of hepatitis B virus (HBV) infection. Acquisition of HBV infection through MTCT is a major public health concern as this carries a 90% risk of progression to chronic HBV infection, leading to liver cirrhosis, end-stage liver disease, liver cancer and premature death (1). Globally, 254 million persons are chronic carriers of HBV (2). The highest prevalence rates are borne by the World Health Organization (WHO) Western Pacific (5.9%) and African (WHO AFRO) regions (7.5%) (3). Of particular concern within the WHO AFRO is the fact that 2.5% of children under the age of five years currently live with chronic hepatitis B despite it being entirely vaccine preventable (3). This disease burden is unacceptably higher than that in any other region in the world, and without urgent intervention, portends derailment of the global progress towards eliminating viral hepatitis as a significant public health threat by 2030 (4).

Among the available strategies for the prevention of chronic HBV infection, hepatitis B vaccination has been recognized as the most effective (5). Universal hepatitis B infant vaccination initiated at 4 or 6 weeks of age, has long been implemented in all 47 WHO AFRO member states (6), achieving over 70% coverage since 2014 (7). Despite this the region maintains the highest burden of chronic HBV infection among under five-year-olds, surpassing the global prevalence of 0.9% (3). The WHO recommendation on hepatitis B birth-dose (HepB BD) vaccination for the prevention of HBV MTCT has been in place since 2009 (5, 8). Further to this, the World Health Assembly in 2016 endorsed the WHO goal to eliminate hepatitis B as a global public health threat by 2030, in part by achieving 90% coverage of timely HepB BD and infant vaccinations (9, 10). Steady progress has been made in the global arena with 115 of 194 WHO member states adopting national HepB BD vaccination programs, although the coverage rate (45%) remains a concern (2, 9, 11). While the Western Pacific region has been able to attain a HepB BD vaccination coverage of 80% in response to its regional burden of disease, the 18% achieved across the 15 WHO AFRO member states that have thus far adopted HepB BD vaccination policies, is a dismal contrast (2, 11, 12).

Recognizing the inequitable implementation and poor program performance of HepB BD vaccination in Africa, several studies have sought to identify what the contributing determinants are (12). These studies note that the sub-optimal program performance is underpinned by a multiplicity of factors including, weak service delivery and inefficiencies across broader health systems, limited skilled health workforce trained to attend to birth and conduct post-natal visits, and the absence of political will to implement the program (10, 12, 13). Previous evidence syntheses on this research focus have relied on limited empirical data from the African region, which tends to provide limited exploration of attendant complex systemic factors (12, 14, 15).

It has been established that complex interventions are likely to have profound system-wide effects which tend to be more evident in weak health systems (16). Petticrew et al. (17), offer a pragmatic approach to conducting robust systematic reviews of complex interventions. Hepatitis B birth-dose vaccination programs meet the definition of a complex intervention on account of the limited degree of flexibility in the timing of administration of the vaccine (within 24 h after birth) to achieve maximum effectiveness, the occurrence of multiple mediators and moderators of effect throughout the program implementation process, and the presence of feedback loops where changes in behavior among the people at the center of the program (including program implementers, external partners and donor agencies, policy- and decision- makers, and end users) encourage further behavioral change and thereby influence the performance and outcomes of the intervention (17, 18). To support rational reforms to existing policy, practice, and future research, we examine if (*and how*) complexity within the health system and / or intervention influence the performance of HepB BD vaccination programs in the WHO AFRO.

## 2 Methods

Using a complexity perspective, an exploratory qualitative systematic review study was conducted in two phases aimed at improving our limited understanding of the interaction between HepB BD vaccination programs and health systems in Africa. The first phase involved a scoping review which then informed the research protocol and execution of the qualitative systematic review in phase 2. The protocol is available at the University of Cape Town repository (https://open.uct.ac.za). Phase 1 was essential in gaining an in-depth and up-to-date understanding of the HepB BD vaccination landscape in the region, highlighting the challenges of its implementation in differing contexts (12). Details of this phase are available in the published scoping review (12). A primary outcome thereof was an adapted systems-based logic model for understanding complexities underlying the implementation of HepB BD vaccination programs (18). The themes derived from the logic model were then used to organize and analyze the findings of this systematic review alongside methodological guidance from Petticrew et al. (17) on conducting systematic reviews of complex interventions. Furthermore, this systematic review adopted the Joanna Briggs Institute (JBI) approach to qualitative synthesis (19) and was conducted in line with the updated Preferred Reporting Items for Systematic reviews and Meta-Analyses (PRISMA 2020) guidelines, see Supplementary File 1 (20).

### 2.1 Literature search strategy

A Boolean search strategy comprising of key search terms and search term synonyms was developed drawing on the target population, intervention, and outcomes. Using this search strategy, peer-reviewed literature was sought from several electronic databases and platforms, namely, PUBMED (including MEDLINE), EBSCOhost (Academic Search Premier, Africa-Wide Information, CINAHL, Health Source: Nursing/Academic Edition, APA PsycInfo), Scopus, and Web of Science (excluding MEDLINE). The complete strategy for each database is provided in Supplementary File 2. Supplementary searches were also conducted by reviewing bibliographies of key articles in order to identify any relevant records that may have been missed by the electronic database searches. Further to this, recommendations on key literature from co-reviewers were obtained and Google Scholar alerts activated to assist in the identification of any upcoming research in the field throughout the review period. Search terms used for Google Scholar alerts included “Hepatitis B birth dose vaccine OR vaccination OR vaccinated,” “Africa OR African,” “deprived country OR countries OR populations.” The final literature search date was 30 September 2022.

### 2.2 Eligibility criteria

Literature sources were included if they met the following criteria: empirical studies of quantitative, qualitative, or mixed methods study designs involving human participants; primary studies conducted in one or more of the WHO AFRO member states; and research exploring HepB BD vaccination as a primary or secondary outcome measure and its complex interactions with the health system. Only articles with accessible abstracts and full texts were included in this review. The search was limited to literature published between 2009–2022, due to the WHO recommendation of universal HepB BD vaccination for all member states since 2009 (8, 21). This time frame ensured relevant and recent literature sources were retrieved and allowed for the observation of country progress in the adoption and implementation of HepB BD vaccination programs. We did not place any restriction on the language of publication in order to lessen the likelihood of language and publication bias, especially given the multi-lingual context within the WHO AFRO. Literature sources were excluded if they were found to, (i) only measure epidemiological outcomes of vaccination; (ii) only investigate hepatitis B infant vaccination administered from 4 or 6 weeks after birth or vaccination programs other than HepB BD vaccination; and (iii) involve research only conducted in non-WHO AFRO member states. Furthermore, reviews, modelling studies, reports and commentaries were excluded from this systematic review.

### 2.3 Literature screening and selection

All search results were imported from the respective databases to Mendeley Desktop^®^ reference manager (22). After removal of duplicates in Mendeley^®^, literature sources were exported to Rayyan^®^, a web-based application for systematic reviews (23). Further duplicates were then detected and resolved. Thereafter title and abstract screening continued in Rayyan^®^, guided by the eligibility criteria. Full texts of studies earmarked for potential inclusion were then retrieved and reviewed for relevance and eligibility. The literature search and screening process was conducted by the primary author (TS-R) and the co-reviewers (EA-D and JO). Where discrepancies arose, a decision was made through discussion and consensus among all reviewers.

### 2.4 Critical appraisal

Following the selection of full texts for inclusion, each eligible study underwent a quality appraisal. Critical appraisal tools developed by the Critical Appraisal Skills Programme (24), the Mixed Methods Appraisal Tool (25) and the assessment scale by Dufault and Klar (26) adapted by Cortes-Ramirez et al. (27) were used as appropriate. The current practice of quality appraisals encourages a description of the judgement of ratings, as opposed to an overall score (24, 25). However, this can be problematic when attempting to report the overall results of multiple appraisal tools applied in a single systematic review. In this systematic review, metrics were developed and used to describe the overall judgement of quality for each study. The Mixed Methods Appraisal Tool scoring was based on the 2011 version (28) and has been used in previous systematic reviews (29, 30). Overall scores were calculated as a percentage of the criteria met (20–100%) (28). In the case of mixed methods studies the percentage of the lowest study component was awarded as the overall score (28). Similarly, we quantified responses to questions in the Critical Appraisal Skills Programme tool (Yes = 1, No = 0, Cannot tell = 0.5) as done in other systematic reviews (31, 32). Overall scores calculated were judged as low-, medium-, or high-quality dependent on their correlated scores within the first-, second- or third- thirds of the total, respectively. The adapted Dufault and Klar assessment scale correlated scores with the overall judgement from low (<5 points) to high (>8 points) relevance (27). Studies considered to be of low quality were not automatically excluded but reviewed and discussed among co-reviewers in order to further evaluate the relevance and value against the quality shortfalls identified. Furthermore, ethical consideration and rigor were assessed by reviewing evidence of author reflexivity and affiliations, transparency on sources of research funding, and declarations of potential conflicts of interest.

### 2.5 Data extraction

The data extraction process was guided by the adapted systems-based logic model tailored to systematic reviews of complex interventions, developed during the preceding scoping exercise, and drawing on the workings of Rohwer et al., on how to make sense of complexity in systematic reviews (12, 18). A study-specific data extraction sheet was designed using this logic model to identify essential variables and interactions within HepB BD vaccination programs, such as: context, intervention design/delivery/execution, and intermediate/health and non-health outcomes (Supplementary File 3). The data extraction sheet provided a standardized systematic record of the data summaries attained from every literature source, ensuring traceability and validity of the data extracted.

### 2.6 Data synthesis and analysis

Descriptive, analytical, and qualitative data extracted from eligible studies were synthesized. Relevance and organization of the data was driven by the theoretical model, and broadly categorized as a feature of implementation, intervention, context, or outcomes. An inductive thematic analysis process was then undertaken with the development of codes and relevant themes. Themes were interpreted for underlying complexities of the intervention or through possible interaction with contextual factors in the intervention causal pathway. In studies where national HepB BD vaccination policies have been adopted, an exploration of both enabling factors and constraints to program implementation was done. In those studies where HepB BD pilot interventions or in-depth inquiries have been conducted in the absence of nationwide program adoption, an exploration of anticipated influential factors was performed.

## 3 Results

**Figure 1:**
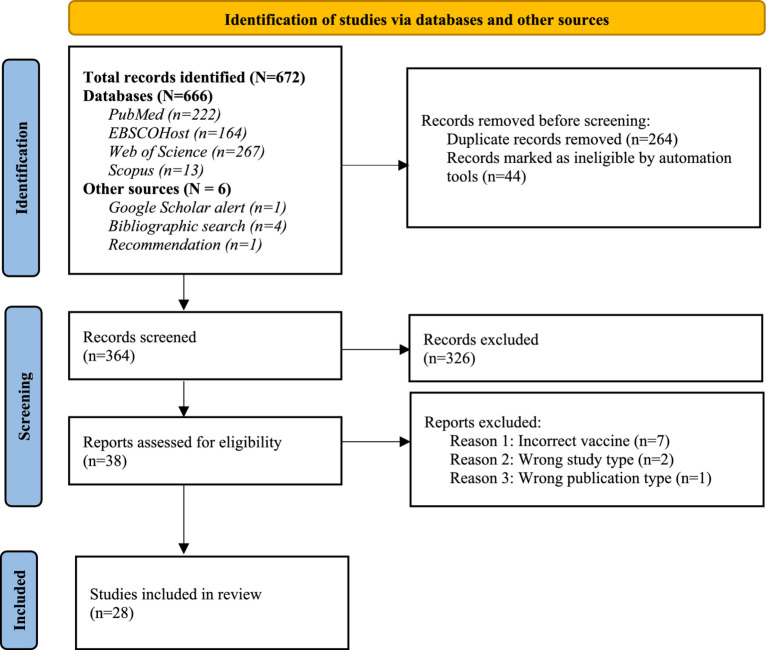
PRISMA flow diagram of the literature search and selection process (Adapted with permission from 20).

The literature search yielded a total of 672 published records. These consisted of 666 articles retrieved via electronic databases, 4 from supplementary bibliographic searches, one from a co-reviewer recommendation and another through a Google Scholar search alert. After deduplication, title and abstract screening, and full text review, 28 articles were judged to be eligible for inclusion in this systematic review, see Figure 1.

### 3.1 Characteristics of included studies

The 28 studies included in this systematic review reported on findings from 12 WHO AFRO member states namely, Nigeria, Senegal, Uganda, The Gambia, Mozambique, São Tomé and Príncipe, Burkina Faso, Ethiopia, Côte d’Ivoire, Benin, Namibia, and Botswana. Included in this tally was one multi-country study reporting findings from five African countries (Nigeria, Namibia, Botswana, São Tomé and Príncipe, and The Gambia) (33). The evidence distribution weighted greatest in Nigeria (*n* = 12), followed by Senegal (*n* = 5). Two studies reported on global findings with results aggregated by region. Of these findings only those relating to the African region were extracted and synthesized. Of the 28 included studies, 26 were published in English and 2 in French. A member of the research team who is a native French speaker worked closely with the primary author (TS-R) to screen, extract and analyze data from these papers with oversight from co-reviewers. A summary of the characteristics of included studies can be seen in Table 1.

**Table 1:** Summary of characteristics of included studies.

Study characteristics (*N* = 28)	Categories among included studies	No. of studies
Country	Nigeria	12
	Senegal	5
	São Tomé and Príncipe	1
	The Gambia	1
	Uganda	1
	Burkina Faso	1
	Mozambique	1
	Côte d’Ivoire	1
	Ethiopia	1
	Benin	1
	Global	2
	Multi-country (Botswana, Namibia, The Gambia, Nigeria, São Tomé and Príncipe)	1
Publication language	English	26
	French	2
Study design	Quantitative cross-sectional	18
	Quantitative cohort	4
	Qualitative	2
	Mixed methods	3
	Quasi experimental	1
Study population	Mother-infant pairs	9
	Infants/children	6
	Mothers	1
	Health care workers	3
	Pregnant women	4
	Countries	2
	Health facilities	3
Primary focus of study	Factors associated with vaccination program performance	14
	Evaluation of broader immunization-related programs	4
	Efficacy of hepatitis B vaccination program regiments	1
	Knowledge, awareness, perceptions, and practice in key populations	9
Vaccination strategy	Universal	20
	Selective	4
	Universal and selective	1
	Not reported	3

Most studies (64.3%; *n* = 18) adopted quantitative cross-sectional designs. The remainder used qualitative (*n* = 2), quantitative cohort (*n* = 4), mixed methods (*n* = 3) and quasi-experimental (*n* = 1) study designs. Based on the methodology, a cross-sectional study was more accurately judged and appraised as having used an ecological study design. One of the qualitative studies included a cost-effectiveness analysis, however for the purpose of this systematic review only the qualitative outcomes were assessed and analyzed. Mothers and mother-infant pairs combined were the largest population group and source of information amongst the studies. Disaggregation of study populations further delineated pregnant women (*n* = 4; 14.3%), mothers (*n* = 1; 3.5%), infants/children (*n* = 6; 21,4%), health care workers (HCWs) (*n* = 3; 10.7%), health facilities (*n* = 3; 10.7%) and countries (*n* = 2; 7.1%). Among those studies involving pregnant women, two were longitudinal studies which provided further information on infants born to these cohorts upon follow-up. Data sources from health facilities and countries included regional experts in the field, informants from the Ministry of Health (MoH), HCWs involved in vaccination services, and partner or stakeholder organizations.

The included studies predominantly focused on identifying factors associated with the performance of HepB BD vaccination programs (14/28, 50%). Of interest, 9/14 (64.3%) studies specifically focused on adherence to the timeliness of HepB BD vaccination of which 7/9 (77.8%) were conducted in Nigeria. A limited number of studies (4/28, 14.3%) evaluated the performance and outcome of broader routine immunization-related programs, with HepB BD vaccination serving as one of several performance indicators. These studies were able to demonstrate vaccine effectiveness in real-life settings. One other study (1/28, 3.6%) determined vaccine efficacy when comparing HepB BD vaccination followed up by two vs three doses of the hepatitis B vaccine in infancy. The remainder were concerned with knowledge, awareness, practice, or perception of HepB BD vaccination (9/28, 32.1%) among key populations such as HCWs (5/9, 55.3%) and pregnant women or mothers (4/9, 44.4%), see the detailed study characteristics in Supplementary File 4. Of the 28 studies, 25% (*n* = 7) were conducted at a time when the relevant countries did not have a national policy for HepB BD vaccination in place. Seventy-one percent of the studies (*n* = 20) were conducted in settings that had implemented universal HepB BD vaccination, while 14.3% (*n* = 4) employed selective HepB BD vaccination, and 10.7% (*n* = 3) did not report their implementation strategy. The multi-country study (*n* = 1) reported on both universal and selective HepB BD vaccination programs in the individual countries investigated.

Regarding the quality of included studies, twenty-one studies were appraised using the Mixed Methods Appraisal Tool (25), six using the Critical Appraisal Skills Programme (24) tool and one using the adapted Dufault and Klar assessment scale (26, 27). Three papers were judged as being of lower quality (Table 2). Notably, those employing a mixed method design were inclined to perform better on the quantitative study components compared to the qualitative ones, which brought down their overall quality ratings. The lowest rated study was a cross-sectional study which did not include details on sample representativeness, or control for possible confounding or modifying factors. None of those judged as low quality were excluded as the data still provided considerable value within context. Nevertheless, overall average scores were high (Table 2).

**Table 2:** Quality appraisal of included studies.

Author, year	Appraisal tool	Overall quality judgement
Accrombessi et al. (2020) (55)	MMAT	
Aina et al. (2017) (43)	MMAT	
Allison et al. (2017) (51)	Dufault and Klar	
Bagny et al. (2015) (45)	MMAT	
Bassoum et al. (2021) (58)	MMAT	
Bassoum et al. (2022) (57)	MMAT	
Chang et al. (2019) (47)	MMAT	
Dagnew et al. (2020) (60)	MMAT	
Djaogol et al. (2019) (44)	MMAT	
Goodman et al. (2013) (49)	MMAT	
Guingané et al. (2020) (54)	CASP	
Hagan et al. (2019) (46)	CASP	
Jaquet et al. (2017) (56)	MMAT	
Loarec et al. (2022) (52)	CASP	
Miyahara et al. (2016) (50)	CASP	
Nankya-Mutyoba et al. (2022) (73)	CASP	
Okenwa et al. (2019) (34)	MMAT	
Okenwa et al. (2020) (36)	MMAT	
Olakunda et al. (2021) (37)	MMAT	
Périères et al. (2021) (53)	MMAT	
Sadoh et al. (2014) (41)	MMAT	
Ibrahim et al. (2022) (38, 39)	MMAT	
Ibraheem et al. (2022) (38, 39)	MMAT	
Ibraheem et al. (2019) (35)	MMAT	
Sadoh et al. (2013) (40)	MMAT	
Danjuma (2020) (42)	MMAT	
Bada et al. (2022) (59)	CASP	
Moturi et al. (2018) (33)	MMAT	

Overall judgement of quality: Mixed Methods Appraisal Tool (MMAT) calculated as a percentage of criteria met, mixed method designs awarded an overall score equivalent to the lowest rated study component of the study; Critical Appraisal Skills Programme (CASP) tools overall scores are calculated and then rated as being in the first, second or last third of the total with the overall judgement correlated as low, medium or high quality respectively; Dufault and Klar assessment scale measures overall judgement of relevance from low (<5 points) to high (>8 points) relevance. In this table: red = low quality; yellow = medium quality; green = high quality.

### 3.2 Sources of complexity in the performance of HepB BD vaccination programs

**Figure 2:**
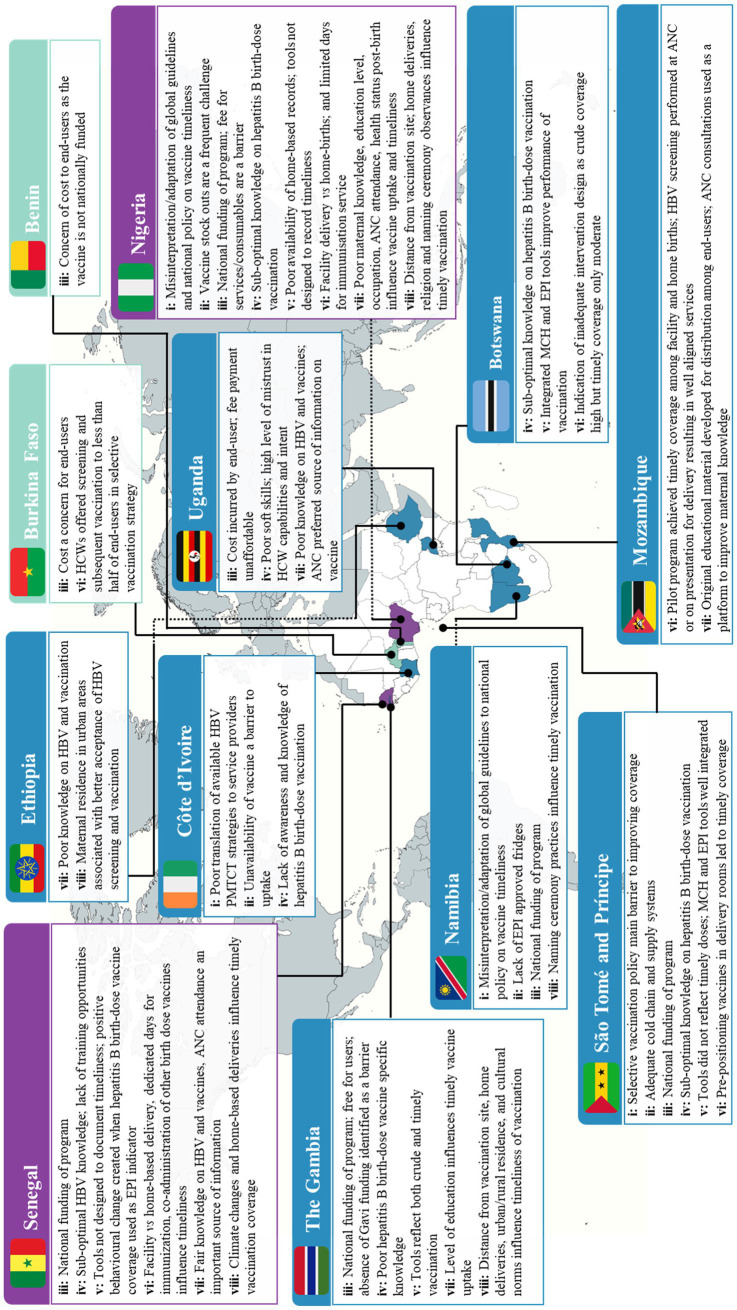
Summary of findings on the sources of complexity influencing the performance of HepB BD vaccination programs in the WHO AFRO. Colour key: ▄ multiple findings across themes ▄ moderate findings across themes ▄ Minimal findings across themes. Theme key: **i**: availability and interpretation of HepB BD vaccination policies; **ii**: capacity of HepB BD vaccine supply and cold chain systems; **iii**: availability of equitable and sustainable financing for HepB BD vaccination programs; **iv**: capacity and capability of HCWs delivering HepB BD vaccination programs; **v**: role of immunization monitoring systems and impaired feedback loops; **vi**: influence of context vs system design on the timeliness of HepB BD vaccination; **vii**: influence of maternal knowledge and socio-economic factors on timely HepB BD vaccination; **viii**: influence of wider contextual factors on timely HepB BD vaccination.

Eight cross-cutting themes were identified across the included studies. These themes describe the complexity found at the intersection of HepB BD vaccination programs and the health systems that deliver them. These eight themes are listed and further unpacked in Table 3. The “influence of wider contextual factors on timely HepB BD vaccination” was the most frequently identified theme (19 of 28 studies) while that on the “role of immunization monitoring systems and impaired feedback loops” was less frequently researched (11 of 28 studies). A summary of the geographic spread of these themes can be seen in Figure 2.

**Table 3:** Frequency of themes identified among included studies.

Theme	Frequency of theme (*N* = 28)	Example of theme
Availability and interpretation of HepB BD vaccination policies	13	National policy on HepB BD vaccination allows for the vaccine to be administered up until 14 days post birth (34)
Capacity of HepB BD vaccine supply and cold chain systems	15	Stock outs ranked 3rd in the reasons for delay in vaccine uptake among mothers (42)
Availability of equitable and sustainable financing for HepB BD vaccination programs	17	Pregnant women expressed concerns about unaffordable cost of the vaccine and charges they may incur should the program be implemented in their country (48)
Capacity and capability of HCWs delivering HepB BD vaccination programs	16	~50% of the medical practitioners surveyed in a study thought it safe to administer HepB BD vaccine at birth (56)
Role of immunization monitoring systems and impaired feedback loops	11	Where vaccination records do not include columns for documenting the time of administration of the HepB BD vaccine, it is difficult to establish timeliness (57)
Influence of context vs system design on the timeliness of HepB BD vaccination	16	Mothers identified the lack of vaccine delivery on Friday evenings, weekends, or public holidays among the major reasons for delayed vaccination (59)
Influence of maternal knowledge and socio-economic factors on timely HepB BD vaccination	18	Maternal level of education up to secondary or higher was positively associated with timely vaccine uptake (35)
Influence of wider contextual factors on timely HepB BD vaccination	19	In the primary health care system in The Gambia, village-based traditional birth attendants and HCWs are supervised by community nurses as more than 40% of deliveries occurred at home (50)

#### 3.2.1 Availability and interpretation of HepB BD vaccination policies

The influence of policy was not explicitly investigated by any of the studies included in this review. Among the 13 studies briefly touching on the direct or indirect role of policy in the implementation of HepB BD vaccination programs, it was important to note the variations in interpreting global recommendations, often resulting in disparate outcomes. The selective vaccination policy in São Tomé and Príncipe was found to be a principal barrier to achieving high HepB BD vaccine coverage rates in the country (33). In studies conducted in Nigeria, guidelines from the National Primary Health Care Development Agency allowed HepB BD vaccination to be administered up until two weeks after birth (34–37). This guideline was open to misinterpretation, likely misleading both HCWs and mothers into assuming the vaccination between day 0 and 14 would infer the same level of protection or effectiveness against HBV MTCT. The average age at HepB BD vaccination in Nigeria ranged across 6 days (38), 12 days (39), 14.3 days +/− 15.6 days (40), and 28.4 days +/− 40.4 days of life (41), with only one study presenting an average age of 1 day (42). In 2015, Nigeria revised its policy on the Expanded Programme on Immunization (EPI) strategies to emphasize timely HepB BD vaccination within 24 h of birth (33). Although well-meaning, this led to further misinterpretation, with HCWs assuming administration of the birth-dose should only be delivered within 24 h or not at all (43). This revised policy led to an overall 30% drop in coverage of hepatitis B vaccinations across 27 health facilities in one study (43). In a later study (conducted between 2017–2018), 1.3% of mothers from Enugu State in Nigeria recommended the HepB BD vaccination policy should mandate vaccination within 24 h to improve timely uptake (34). Also noteworthy was a similar case of policy misinterpretation in Namibia where the national recommendation on HepB BD vaccination allowed for administration up until 2 weeks post birth (33), although further insights were not provided on the performance of the program in the context of this local policy.

Plausible reasons for the misinterpretation of hepatitis birth-dose vaccination policies at the national level may be drawn from the influence of other birth-dose vaccination policies, such as those for Bacillus Calmette-Guérin (BCG) and oral polio vaccine (OPV). In instances where guidelines state that the birth-dose of OPV should be administered before 2 weeks of life (40) and BCG before 12 months of age (35) it was observed that HCWs in some countries tended to group birth-doses, leading to delays in administering the HepB BD vaccine. Accordingly, in a study conducted by Ibrahim et al. (39), it was suggested that the 14-day policy on administering BCG and OPV birth-doses affects the timely receipt of the HepB BD vaccine in Nigeria, as HCWs often wait to administer them together. In Senegal however, a study exploring the perspective of HCWs on the acceptability and perceived challenges of implementing HepB BD vaccination found that they demonstrated good understanding of the need to vaccinate, the health benefits and the recommended timing (44). One HCW described their approach to home birthed neonates, grouping those presenting before and after 24 h post birth (44). This interpretation of the policy by the HCWs acknowledges their understanding of the time sensitive nature of HepB BD vaccination (44). To the contrary, midwives in Côte d’Ivoire cited “ignorance” on available HBV MTCT prevention strategies as one of their reasons for not administering the birth-dose vaccine (45).

Only two of the included studies addressed the importance of written guidelines or standard operating procedures at the health facility level, especially in instances of vaccinating premature or very low birth weight (VLBW) infants. This includes a study by Moturi et al. (33), which noted that only 26% of facilities in Nigeria and 36% of facilities in Namibia had written protocols, whereas the five health facilities (of differing levels of care) studied in São Tomé and Príncipe did not have any available (46). High HepB BD vaccine coverage rates observed in a Nigerian private hospital was associated with adopting facility guidelines in the form of a discharge checklist which included a HepB BD vaccination check in place (33).

#### 3.2.2 Capacity of HepB BD vaccine supply and cold chain systems

Survey respondents participating in a global study conducted by Chang et al. (47), proposed improvements in vaccine supply, delivery, and storage as an approach to enhance global prevention of hepatitis B. As part of this survey study, local experts addressed the need to improve access to vaccines in hard-to-reach areas in Africa to reduce untimely administration of the HepB BD vaccine to neonates (47). In line with this, interrupted vaccine supply or stockouts were identified by mothers and pregnant women participating in six other studies as contributors to delayed vaccine uptake (34, 38–40, 42, 48). Only two of the six studies rated stockouts as a major reason for delayed birth-dose vaccination (34, 42). Similarly, in three additional studies, HCWs found that unreliable vaccine supply chains, specifically vaccine stockouts, were limitations to the successful implementation of the HepB BD vaccination programs in their settings (44, 45, 49).

While vaccine stockouts have had a negative impact on HepB BD vaccination programs in Africa, improvements in the supply chain have been noted in the region. Only two of 78 facilities investigated across African countries reported experiencing stockouts in one multi-country study (33), whereas findings from another study reported the duration of stockouts lasting less than 2 weeks (46). Multiple studies included in this review indicate that vaccines are supplied by central government (35, 39, 46, 50) in a process coordinated by state or regional health teams. A central area or depot is then accessible for the collection of vaccines to districts and facilities (35, 39, 50). Two studies conducted in Nigeria for example, noted that collection from these central areas by vaccinators take place 2–3 times a week (35, 39). In Kano State, Nigeria, a direct-to-facility-delivery approach was trialed (43). This initiative resulted in a statistically significant decrease in vaccine stockouts and an increase in stock adequacy levels due to reduced bottlenecks at the local government authority cold stores (43). The HCWs participating in this study reported being able to afford more time for direct patient care and health facility management, and less time away from their posts when collecting vaccines (43). A year after the initial roll-out and implementation of the initiative, an increase in vaccine coverage was noted with positive results in the coverage of nearly all vaccinations monitored in Kano (43). However, coverage of the HepB BD vaccine decreased owing to a misinterpretation of the national policy as described previously (43). Explicit information on vaccine supply to private health facilities was not available from the included studies, although one study reported on the exchange between Namibian and Nigerian private facilities and their respective MoH, where vaccine supply was received in exchange for monthly reports including coverage data (33).

Regarding adequate and sustainable storage, it has been noted across the evidence-base that power outages influence the functioning of cold chain systems, leading a HCW in Senegal to suggest the use of solar energy as an alternative power source (44). Accordingly, a study conducted in The Gambia reported the use of solar panels to operate vaccine fridges (50), although no details were provided on whether this improved the cold chain system. The matter of EPI approved fridges was mentioned in two studies as either absent or working well. Moturi et al. (33), note that 52% of Nigerian and 12% of Namibian facilities studied lacked EPI approved fridges while facilities in São Tomé and Príncipe, Botswana, and The Gambia were found to have good quality cold chain systems. Further to this, all five facilities in São Tomé and Príncipe assessed by Hagan et al. (46), possessed EPI approved fridges with vaccines being monitored twice a day.

#### 3.2.3 Availability of equitable and sustainable financing for HepB BD vaccination programs

In-depth exploration of the funding mechanisms for HepB BD vaccination programs was largely absent from the included studies. In one study assessing 62 countries including 13 WHO AFRO member states, it was revealed that 55% had their HepB BD vaccines covered by government funding and 5% by private insurance (47). Miyahara et al. (50), addressed the lack of funding support from Gavi, the Vaccine Alliance, for HepB BD vaccination programs compared to the pentavalent vaccine in eligible countries within Africa. It has been reported that increased national health expenditure *per capita* correlates with higher HepB BD vaccine coverage rates (*p* = 0.03), highlighting the need to strengthen domestic investments to supplement support from external sources (51). In the study assessing direct-to-facility vaccine supply for example, the initiative was reported to have been funded by a tripartite agreement involving the Bill and Melinda Gates Foundation, the Dangote Foundation, and the Kano State Government of Nigeria (43). The inclusion of state funding was aimed at encouraging greater political will and country ownership (43). Similarly, a HepB BD pilot program in Mozambique was reported to have received funding from Médecins Sans Frontières (Doctors Without Borders) in partnership with the national MoH (52).

Where financial accessibility of the HepB BD vaccine is concerned, seven studies reported that HepB BD vaccination was free for users as part of their national immunization schedule, particularly in public health facilities (33, 35, 38, 44, 46, 47, 53). Moturi et al. (33), noted the existence of fee payments for HepB BD vaccination in private facilities in Botswana and Nigeria. End users were required to pay a fee to cover the cost of services, in some instances due to consumable shortages, despite the vaccine itself being free (33, 34, 49). In a quasi-experimental study conducted in Nigeria investigating the perceptions of mothers before and after HCW sensitization, 80% of respondents judged the fees charged to supplement shortages in consumables to be reasonable (49). However, in another study conducted in Nigeria, 6.3% of mothers participating in a study reported fee payment for immunization as their reason for delayed vaccination (34). Some mothers (8.1%) in this study recommended the vaccine should be entirely free of charge in order to improve timely uptake in Enugu State, south-east Nigeria (34). In Uganda pregnant women residing in both urban and rural settings believed the cost of the HepB BD vaccine to be unaffordable, and raised concerns of charges it would carry when implemented (48). Similarly, the pilot study on prevention of MTCT (PMTCT) of HBV in Burkina Faso, reported the cost of the HepB BD vaccine to be 7.76 USD, incurred entirely by the consumer (54). Other costs borne by the consumer included those for HBV screening tests, treatments, and vaccination (54). The authors acknowledge that the costs of all tests, treatments, screening, and vaccines need to be considered in relation to the income levels in Burkina Faso (54). In Benin, Accrombessi et al. (55), also elaborate on the high out-of-pocket expense of HepB BD vaccination, costing 8 USD, given that the vaccine had not been included in the national immunization schedule at the time of the study being conducted. In this same study, it was reported that HCWs recommended HepB BD vaccination to mothers according to their financial means (55). No further details were provided on how HCWs in this study assessed parents’ financial capabilities prior to recommending the HepB BD vaccine (55).

#### 3.2.4 Capacity and capability of HCWs delivering HepB BD vaccination programs

A dominating theme within the included studies was the lack of training for vaccinators or other HCWs involved in HepB BD vaccination programs. Two main population groups offered valuable insights on this, end users (mothers or pregnant women) (34, 49), and HCWs themselves (33, 40, 44–47, 56). In the qualitative component of a mixed method study conducted in Senegal, overall attitudes and beliefs among HCWs on HepB BD vaccination was judged to be good (44). However, generally HCWs lacked basic knowledge on HBV and had limited access to HepB BD specific training, although 85% knew the first dose should be administered within 24 h of birth (44). Interestingly, in this same study, those predominantly involved in vaccination services (46%) were more likely to have middle or lower levels of formal education (72%) but were also more likely to have received HBV-specific training (72%) (44). Of those who were mainly involved in antenatal care (ANC) and activities (54%), only 47% had received HBV-specific training (44). In a São Tomé and Príncipe study, 80% of facilities received training on HepB BD vaccination and HCWs in all five sites were aware that administration of the birth-dose should be within 24 h post birth (46). A study conducted to assess the knowledge and attitude among medical practitioners working in an urban setting in Senegal, reported that 21% attained low HBV knowledge scores (56). Among these medical practitioners, a low level of knowledge was attributed to not attending any HBV-specific lectures after basic medical training (odds ratio or OR 6.0 [95% confidence interval or CI 1.4–26.4]) (56). Among the total population of medical practitioners studied, only 51.2% thought it safe to administer vaccines to newborns, of which the rest linked misconceptions of infertility (48%) or neurological disorders (37.8%) to the vaccination (56). In a multi-country study, the lack of training for HCWs specific to HepB BD vaccination ranged from 56% in The Gambia to 88% in Botswana (33). Knowledge of the recommended administration window was high but suboptimal knowledge of contraindications and age-limits were noted (33). False contraindications reported included prematurity, VLBW, and acutely ill but stable infants (33). Additionally, breastfeeding was delayed and discouraged by HCWs according to a São Tomé and Príncipe study until after the HepB BD vaccine was administered for fear of HBV MTCT (33). Similar findings were observed in another São Tomé and Príncipe study where health centers were less likely to vaccinate VLBW, premature, or clinically unstable neonates (46).

In Senegal, immediate hospitalization of neonates was significantly associated with poorer outcomes of timely HepB BD vaccination (adjusted odds ratio or AOR 0.42, [95% CI 0.26–0.68]), whereas weighing neonates increased the chances of timely vaccination (AOR 3.90, [95% CI 1.79–8.53]) (57). Both these practices could be related to the lack of knowledge on contraindications, and the confidence to vaccinate only when the infants’ weight suggests a better perceived assessment of health. An alternate and plausible explanation offered relates to reluctance among HCWs to vaccinate hospitalized or VLBW neonates in order to avoid any adverse events being linked to the vaccine or the vaccinators themselves (57). In contrast, a related study in Senegal found that immediate hospitalization of neonates after birth increased the odds of benefitting from co-administration of birth-dose vaccines by 1.74 times, when compared to those not requiring hospitalization after birth (AOR 1.74, [*p* = 0.002]) (58). Weighing the newborn was also associated with better chances of co-administration of birth-dose vaccines (*p* = 0.006) (58). Miyahara et al. (50), discuss the need to improve HepB BD vaccination awareness and training among delivery agents in the Gambia as no difference in timing was found between those delivered in health facilities and those born elsewhere. Similarly, in a Nigerian study, despite frequent contact with the health care system (92.2% of pregnant women attended ANC consultations and 81.1% delivered in a health facility) only 57.7% of women cited receiving information from HCWs on when to commence HepB BD vaccination (40). Furthermore, sources of information on HepB BD vaccination were further disaggregated into nurses (28.1%), ANC visits (20.3%), immunization sessions (17.2%), doctors (1.6%), unspecified HCWs (4.1%) and unspecified hospital activity (10.6%) (40). This finding supports that of a study conducted in Uganda where mothers reported that HCWs provided limited communication regarding vaccination needs, discouraging their involvement in the program (48). In two HBV PMTCT pilot programs conducted in Burkina Faso and Mozambique, training of HCWs on HBV prophylaxis, screening, counselling, and vaccination was conducted prior to rolling out the program (52, 54). In Mozambique, Loarec et al. (52), indicate that training was given to project nurses, MoH nurses and midwives alike, consisting of a 1-day training course or on-the-job training. Despite training of HCWs on HBV prophylaxis, screening, and counselling in the Burkina Faso pilot program, less than half of the pregnant women accessing services during this pilot were offered hepatitis B screening (54). Sub-optimal screening practices led to missed opportunities to identify and timely vaccinate at-risk neonates. Reasons for poor HBV screening and targeted birth-dose vaccination practices reported by midwives in a study conducted in Côte d’Ivoire, include lack of awareness, lack of time, increased workload, and unavailability of vaccines (45). Consequently, 41.4% of midwives reported not performing screening, while 52.3% reported not administering HepB BD vaccination to HBV exposed newborns (45). To mitigate such practices, a study in Nigeria trained HCWs by sensitizing them to improve the quality of immunization services (49). Post-intervention, a significant increase was found among mothers of the study group across two criteria; those who found information provided to them on immunization adequate; and those who correctly identified the number of visits left to complete the immunization schedule (49).

Regarding non-technical skills and communication of HCWs, younger pregnant women in Uganda viewed HCWs as rude and uncaring (48). They were also described as often not providing important information about newborns, including communication on vaccination requirements (48). This discouraged the buy-in of mothers and limited demand for the HepB BD vaccine, leading to missed opportunities for vaccination (48). Delayed vaccination was also linked with mistrust of HCWs (48). Pregnant women expressed concern about HCWs handling their newborns and administering injectables (48). They instead preferred oral vaccines over injectables as it reduced the risk of HCWs making errors when administering vaccines (48). The quasi-experimental study in Nigeria on the other hand, found that pre-intervention, 80% of women in both study and control groups felt that HCWs treated them with respect, were approachable and polite (49). Further to this, a statistically significant increase was observed among respondents in the study group post-intervention who rated HCWs approachable (*p* < 0.05) (49). Lastly, another aspect of the capability of HCWs explored in the evidence-base was the importance of delegating duties. Across facilities studied in five African countries, senior oversight by medical practitioners was not required in order to deliver HepB BD vaccination, allowing midwives and other qualified cadres of HCWs to administer vaccinations without undue delays (33).

#### 3.2.5 Role of immunization monitoring systems and impaired feedback loops

Of the 28 included studies, 11 touched on data collection and information systems, with multiple studies referring to reliance on vaccination cards to monitor the administration of HepB BD vaccines (33, 34, 36, 37, 41, 46, 50, 52, 53, 57, 58). The monitoring process should serve as an active feedback loop, encouraging improvements as vaccine coverage outputs guide future operations of the program. However, if monitoring systems are inaccurate or data collection tools are inadequately designed (i.e., not fit for purpose), feedback loops are unlikely to be effective in improving programs and broader systems functioning. In the HBV PMTCT pilot program in Mozambique, reference to the suboptimal quality and completeness of data was accepted as a characteristic of the real-world setting (52). The dilemma in monitoring the accuracy of HepB BD vaccination coverage was recurringly linked to the reliance on home-based immunization records. In studies conducted in Nigeria, only 27.3% of children had their immunization cards available in one study (41) while 44% of mothers in another offered verbal confirmation of vaccination due to unavailable or ambiguous home-based records (37). The reliability of vaccination history recall is of course questionable as was demonstrated in a study conducted in Senegal which noted disparities between HepB BD vaccination coverage based on reports by mothers/caregivers versus that recorded in home-based or facility records (57). Overall, vaccination coverage reported by mothers/caregivers in this study was approximately 10% less than that recorded in home-based or facility vaccination records (57). In another study conducted in Senegal, the availability of home-based records was found to be associated with high co-administration rates of birth-dose vaccines, with those having home-based records reported to be 6.88 times more likely to receive co-administered birth-dose vaccines compared to those without (AOR = 6.88, [*p* = 0.006]) (58).

Health facility records have frequently been used to correlate the accuracy of vaccination coverage or to determine the timeliness of HepB BD vaccine administration (34, 36, 57). Again, in Senegal, Bassoum et al. (57), found that HepB BD vaccine coverage rates were largely concordant between home-based records (82.3%) and health facility registries (84.1%), with similar trends noticed in coverage of other birth-dose vaccines. However, in the absence of columns dedicated to documenting the time of vaccination, establishing timeliness required calculation of the difference between the date of birth from health facility records and the date of HepB BD vaccine administration (34, 36, 57). This was instrumental in determining the large discrepancy between crude HepB BD coverage (88.5%) and valid timely doses (42.1%) (57). In São Tomé and Príncipe, although all 5 study sites provided written documentation, the date of HepB BD vaccination was not recorded and therefore establishing timeliness was not possible (46). Practices among facilities in The Gambia included adapting EPI records to reflect both timely and crude HepB BD vaccination (33). These studies underscore the need for clear and appropriate policies and guidelines, without which information systems cannot be designed to be fit for purpose, disrupting feedback processes, and rendering data, like vaccine coverage and timeliness less useful, for appropriate action.

In the multi-country study conducted by Moturi et al. (33), facilities in all five participating countries (Nigeria, Namibia, Botswana, São Tomé and Príncipe, and The Gambia) reported having designated columns for recording HepB BD vaccination in their EPI reporting and recording tools, although older versions of these tools (without these columns) were still circulating in some facilities. In addition, tally sheets and reporting forms at facilities were routinely updated, but none of the maternity registers were modified with columns to record receipt of HepB BD vaccines (33). In health facilities in São Tomé and Príncipe, maternal child health (MCH) and the EPI tools were integrated, facilitating collaboration on implementing the HepB BD vaccination program (33). Similarly, health facilities in Botswana recorded data on HepB BD vaccination in both EPI tools and delivery registries (33). One study described a possible knock-on effect of monitoring, where timely HepB BD vaccination was used as an EPI performance indicator and may have encouraged better timely coverage of the vaccine when compared to other birth-dose vaccines in the study (BCG = 13.9%; OPV = 30%; HepB BD vaccine = 42.1%) (58).

#### 3.2.6 Influence of context vs system design on the timeliness of HepB BD vaccination

Twelve of the included studies explored timeliness of HepB BD vaccination. Even among those studies where measurement of timely vaccine administration or factors associated with delayed HepB BD vaccination were not the primary focus, timeliness emerged as an important challenge. Frequently reported determinants of timely vaccination among the studies reviewed ranged from institutional deliveries and health facility type, inaccessibility of immunization services and vial dosage and co-administration of birth-dose vaccines. Across the evidence-base, a valid or timely dose was often defined as vaccination on the day of birth or the day thereafter. However, time frames used to assess timeliness of HepB BD vaccination differed across countries and studies but frequently fell within day 0–1, day 0–7, and day 0–14, although in a study conducted in The Gambia, birth-dose vaccinations were recorded even after 6 and 12 months after birth (50). The summarized data presented in Table 4 reflects two key findings on the timeliness of HepB BD vaccination in the region; (i) that the vaccination was typically recorded between days 0–1 or 0–7 after birth with wide coverage rates ranging between 1.1% – ~92.4%, and (ii) that generally, vaccine coverage rates tend to increase with increasing age, with the highest rates frequently recorded from day 0–14 and over.

**Table 4:** Timeliness and coverage of the HepB BD vaccine in WHO AFRO member states.

Study (Author, year)	Country	HepB BD vaccination coverage (%)							^ƚ^Median/mean age at receipt of vaccine
		Crude	*Day 0–1	Day 0–7	Day 0–14	Day 0–28	6 m	12 m	
Bassoum et al. (2022) (57)	Senegal	88.1	42.1	–	–	–	–	–	–
Périèras et al. (2021) (53)	Senegal	71.5	54.4	58.2	–	–	–	–	–
Okenwa et al. (2019) (34) and Okenwa et al. (2020) (36)	Nigeria	–	26.2	–	–	–	–	–	–
Ibrahim et al. (2022) (39)	Nigeria	–	11	26.3	68.5	–	–	–	12 days
Miyahara et al. (2016) (50)	The Gambia	–	1.1	5.4	–	58.4	93.1	93.3	24 days
Loarec et al. (2022) (52)	Mozambique	83.4	89.4	–	–	–	–	–	–
Guingane et al. (2020) (54)	Burkina Faso	–	78.3	–	–	–	–	–	–
Sadoh et al. (2014) (41)	Nigeria	–	–	31.7	39.0	43.9	–	–	28^ƚ^ days ± 20.4 days
Ibraheem et al. (2022) (38)	Nigeria	75.1	20.5	~52.4	~68.1	~79.4	–	–	6 days
Sadoh et al. (2013) (40)	Nigeria	–	1.3	43.1	70.6	89.5	–	–	9 days
Danjuma et al. (2020) (42)	Nigeria	–	~53.8	~92.4	-	-	–	–	1 day
Ibraheem et al. (2019) (35)	Nigeria	~100	~49.8	~87.8	~94.6	~100	–	–	2 days
Bada et al. (2022) (59)	Nigeria	99	33	91	–	–	–	–	–
Olakunde et al. (2021) (37)	Nigeria	53	–	–	–	–	–	–	–
Moturi et al. (2018) (33)	The Gambia	84	7	–	–	–	–	–	11 days
	Nigeria	23	13	–	–	–	–	–	–
	Botswana	94	74	–	–	–	–	–	–

*Defined as vaccination on the day of birth or the day thereafter. – Timeframe not recorded. ~ Approximation of HepB BD vaccination coverage value as disaggregated coverage among the 3 birth-doses (OPV, BCG, HepB BD) was not available in the respective study. ^ƚ^Mean age at receipt of vaccine.

Ibraheem et al. (38), report that in Nigeria, the HepB BD vaccine performs the poorest when comparing crude coverage rates of all three birth-doses: 75.1% vs. 91.2% for BCG and 82.1% for OPV, respectively. More importantly, this study observed that 20.6% of infants presented for all three birth dose vaccinations beyond day 28, with the majority (78.8%) not presenting on day 0–1 (38). This finding is in line with those from previous studies conducted by Sadoh et al. (40), which showed poor adherence to timely birth-dose vaccinations in Nigeria, where only 1.3% of neonates presented within 24 h of birth in one study and 56.1% of children received their HepB BD vaccine beyond day 28 in the other (41). In contrast, two other studies conducted in Nigeria reported better compliance to timely administration of HepB BD vaccination as the majority in one study presented (49.8%) within one day after birth and only 5.4% of infants beyond 14 days (35), while in the other study, 53.8% of infants received their birth-dose vaccinations within 24 h after birth with nearly a third presenting between day 1–7 (42). Despite reporting the highest crude coverage estimates (98%) of HepB BD vaccination, the Kweneng District of Botswana in fact had the lowest timely estimates (62%) compared to other districts in the country (33). A noteworthy knock-on effect of untimely HepB BD vaccination is the further delay in uptake of hepatitis B infant vaccination, as highlighted in studies from Senegal, Nigeria and globally (41, 47, 53).

The influence of institutional delivery on access to the HepB BD vaccine emerged as a prominent sub-theme under timely administration. Institutional delivery rates in WHO AFRO was positively and significantly associated with optimal coverage of the HepB BD vaccine (rho = 0.89; *p* = 0.04), as reported by Allison et al. (51). More specifically, among other included studies, seven found an association between institutional delivery and timely administration of the HepB BD vaccine (33, 35, 36, 38, 40, 53, 57). Neonates in a Senegalese study where most pregnant women (68.8%) delivered at a health facility, were 1.62 times more likely to receive timely HepB BD vaccination compared to their counterparts who were born elsewhere (AOR 1.62; [*p* = 0.046]) (57). In Nigeria, hospital delivery increased the odds of timely vaccination by 6-fold (OR 6.36, [95% CI 1.33-30.38]) (35) and was a determinant of vaccination by day 0–1 compared to those presenting after day 1 (35). Despite most mothers (95.1%) delivering at a health facility in another Nigerian study, only 26.9% of the infants studied were administered timely doses, however the authors still observed a significant association between delivery at a health facility offering immunization services and the timely receipt of the HepB BD vaccine (36). This was advantageous to those delivering at such a health facility compared to those who did not (AOR 5.39, [95% CI 2.45-11.87]) (36). Another study used a 1-week metric and reported that 50% of those delivering at a health facility and only 20.7% of those delivering outside of health facilities presented within this time frame for birth-dose vaccination (40). Though not a statistically significant finding, Bassoum et al. (58), found high facility delivery (71.8%) in Senegal to be an enabling factor for the co-administration of birth-dose vaccines. Similarly in another Senegalese study, being born at home as opposed to a health facility was significantly associated with non-adherence to timely administration of the HepB BD vaccine at the 10% threshold (AOR 2.02, [*p* = 0.077]) (53). Furthermore, HCWs in Senegal who were interviewed as part of a study by Djaogal et al. (44), expressed their view of home deliveries being a barrier to timely vaccination and suggested sensitizing women to give birth in health facilities.

When stratified by health facility type, public facilities were favored over private ones where timely administration of the HepB BD vaccine was concerned (36, 42). Danjuma et al. (42), for example, found that private health facilities in North-Central Nigeria were more likely to delay HepB BD vaccination by 2-fold compared to public health facilities [AOR 2.616; *p* = 0.003]. Another Nigerian study investigating the influence of the place of birth on the receipt of the HepB BD vaccine among 12-24-month-old children found the odds of vaccination were low in private facilities (AOR 0.77, [95% CI 0.59-0.99]) and home deliveries (AOR 0.48, [95% CI 0.36-0.63]) (37). Further to this, the odds of vaccination among neonates delivered at home when compared to those delivered at a private health facility was also found to be significantly lower in this study (AOR 0.62, [95% CI 0.43-0.88]) (37). Among reasons offered by mothers for delayed vaccination, 8.5% listed having delivered at a private hospital, 3% having delivered at home and another 3% delivered at church (35). In comparison, 91.3% of mothers participating in the study by Okenwa et al. (34), identified the unavailability of the vaccine at the delivering facility more than the actual place of delivery as the reason for delayed vaccination. In this study, 95.05% of mothers delivered at health facilities, with the majority delivering at private health facilities (53.5%) and public primary level care facilities (24.7%), but only 63.77% delivered at a place where the vaccine was offered, inferring that birthing facilities did not always offer birth-dose vaccination (34). Contrary to the aforementioned studies, two other studies found minimal influence of the place of delivery on the timely administration of the HepB BD vaccine (50, 52). In the Gambia, while 59.7% of neonates were delivered at a health facility, only 0.6% had been vaccinated by day 1 and 3.8% by day 7 (50). Such coverage rates were not much higher than those recorded for the 40.3% of infants delivered outside of health facilities (day 1 = 1.3%; day 7 = 5.2%) (50). Similarly, comparable coverage rates of timely vaccination between home births (80%) and facility delivery (75.4%) were recorded in the HBV PMTCT pilot program in Mozambique, although the proportion delivering at home (*n* = 5) was much lower than those who delivered in health facilities (*n* = 199) (52). It is important to note, that during this pilot program, follow-up processes were integrated into routine ANC consultation where women who missed appointments were contacted by phone and those presenting for delivery were screened and their HBV exposed infants vaccinated as soon as possible by midwives (52).

Another key sub-theme was the accessibility of immunization service and its influence on timely uptake of HepB BD vaccination. This emerged across findings from 11 included studies (33–35, 38–40, 42, 50, 53, 57, 59). Most frequently cited as a barrier to accessing timely HepB BD vaccination was the allocation of immunization services on certain days of the week. In Nigeria, vaccination services were reported to only be available from Monday to Friday, excluding weekends and public holidays (35, 38, 39), or on Tuesdays and Thursdays in other facilities (42, 59). Exceptions were made when the number of deliveries were large enough to warrant vaccination on days other than the two routine vaccination days (42). In relation to this, mothers across six studies identified the lack of vaccine delivery on Friday evenings, weekends, or public holidays among the major reasons for delayed vaccination (38–40, 42, 59). Further reasons proffered by mothers for delayed vaccination included having fixed days for immunization clinics (4.2%) (35), not delivering (75.6%) (34) or presenting on a routine facility immunization day (31.2%) (42), being given a later date to return for vaccination (11.2%) (39) or waiting for the day of BCG immunization services (30.3%) (40). In a study by Ibrahim et al. (39), where vaccination services were available Monday to Friday from 8:00 – 15:00, delivery on specific days of the week was not found to have any statistically significant association with timely receipt of the HepB BD vaccine.

Further to the discourse on service accessibility, other studies provide useful insights into how the design of broader services influence the performance of HepB BD vaccination programs within the African region. In The Gambia, reproductive and child health clinics responsible for vaccinations take place once or twice a week, and a set schedule of supplementary outreach clinics take place on the other days of the week (50). Périères et al. (53), report that four health care posts found in the most rural areas in Senegal provide vaccination services on a particular day of the week and offer outreach to the villages furthest from the post. This contrasts with the situation across the five health facilities studied in São Tomé and Príncipe where daily birth-dose vaccination services were routinely offered without any supplementary outreach services (46). High timely HepB BD vaccination coverage was recorded in São Tomé and Príncipe, particularly among facilities that store HepB BD vaccines in labor wards (33). This was confirmed by findings from Hagan et al. (46), where health facilities in São Tomé and Príncipe provided HepB BD vaccination in delivery rooms. Maternal recommendations for improving timely vaccination in a Nigerian study echoed these insights, suggesting pre-positioning vaccines in labor rooms (22.7%) and making the vaccine available at all birthing health facilities (14.8%) (34). In addressing the design of services and wider systems, it is also important to highlight the role of vaccine technologies. Of the 28 studies, four addressed the use of multi-dosage vials for the three commonly administered birth-dose vaccines within the WHO AFRO (38, 39, 50, 57). Hepatitis B birth-dose vaccines supplied in a 10-dose vial are valid for use up to 4 weeks once opened under the correct storage conditions (57). In contrast, BCG is supplied in 20-dose vials and only valid for use for up to 6 h after opening (57). As such, vials are unlikely to be opened unless 10–12 neonates present for vaccination. Should they be born on a day BCG is not administered, they are unlikely to receive the BCG vaccine on day 0–1 as reported by a study conducted in Senegal (57). This was considered as one of the contributing factors to the better performance of HepB BD vaccination compared to BCG and even OPV (42% vs 13.9 and 30%, respectively) in this study (57). However, these practices may limit feasibility of timely administration at the time of delivery as found in Miyahara et al. (50), where multi-dose vials were a barrier to integrating all three birth-dose vaccination programs within broader maternal and neonatal health services.

#### 3.2.7 Influence of maternal knowledge and socio-economic factors on timely HepB BD vaccination

Maternal factors emerged as a prominent theme across the included studies. These factors included maternal awareness and knowledge of HBV and vaccination, ANC attendance, the health and well-being of mothers and infants’ post-birth, maternal level of education, maternal occupation, and maternal wealth. In terms of maternal awareness and knowledge of HBV and hepatitis B vaccination, this was found to have a statistically significant effect on the adherence to timely receipt of the HepB BD vaccine as demonstrated in three studies (35, 36, 40). Among them, timely vaccination was 2.4 (AOR 2.36, [95% CI 1.38–4.03]) (36) and 3 (OR 3.06, [95% CI 1.16–8.23]) (35) times more likely among infants born to mothers with good overall knowledge on HBV and vaccination. In a study focusing on the co-administration of birth-dose vaccines, knowledge of co-administration and vaccine timeliness among mothers was found to be associated with better co-administration rates, it also predisposed neonates to receive all birth-dose vaccines on the same day (58). Studies surveying maternal reasons for delayed presentation frequently identified the lack of knowledge on the timing of vaccination (34, 38, 40). In one of these studies, poor knowledge, and awareness on the timing of vaccination was the third highest reason for delayed presentation as cited by 72.8% of mothers (34). In support of this trend, findings from Ethiopia demonstrated that 89.6% of pregnant women attained poor overall scores on HBV knowledge, performing poorly in categories on the viral origin (87%), MTCT (87%) and the existence of a vaccine (85%) (60). Similar themes emerged from a qualitative study conducted in Uganda which found sub-par knowledge among pregnant women participating in focus group discussions, contributing to their poor overall understanding of HBV and vaccination (48). A notable observation was that both these studies were from countries yet to adopt HepB BD vaccination as part of their EPI. In Senegal and Nigeria where the HepB BD vaccine is part of the EPI, mothers were found to perform well when assessed on their knowledge of commencement of the vaccination schedule, the benefits and co-administration of birth-dose vaccines (58), vaccine timeliness, HBV MTCT, and disease awareness (36).

Additional factors influencing maternal knowledge and awareness of HepB BD vaccination include place of residence, access to media, improved socio-economic status, gravidas, and level of education. In Nigeria, residing in rural areas was a negative predictor (AOR 0.55, 95% CI [0.34-0.89]) of good maternal awareness and knowledge of HBV (36), whereas in Senegal, mothers with access to television were 1.7 times more likely to receive timely HepB BD vaccination compared to those without (57). This was likely due to better access to information and information sharing mediums (57). Dagnew et al. (60), further showed that in Ethiopia, increased monthly income and primigravida were positively associated with good HBV knowledge; those earning >4,000 Ethiopian Birr were 3.2 times more likely to have good HBV knowledge than those earning <2000 Ethiopian Birr, and primigravidae were 2.9 times more likely to have good HBV knowledge than multigravidas. In addition, maternal education at both primary and secondary levels was associated with good HBV knowledge in this study (60). Good knowledge of HBV was then further associated with better attitudes towards HBV treatment, screening, and vaccination, as 57% of pregnant women were willing to have their babies vaccinated against HBV while 53% demonstrated favorable attitudes toward vaccination, screening, and hepatitis B treatment (60). A Nigerian study also demonstrated the positive effect of maternal tertiary education (AOR 2.10, 95% CI [1.28-3.46]) on good maternal knowledge and awareness of HBV (36). In Senegal, HCWs found that mothers or pregnant women tend to experience difficulties in understanding the concept of HBV when they had no formal education (44). A survey of global experts suggests inclusion of education on hepatitis B in public education campaigns with the aim of increasing public awareness and motivation to vaccinate (47). Accordingly, as part of the pilot program in Mozambique, original educational material was developed as well as advice given during screening to improve knowledge and awareness among women (52). This practice supports maternal recommendations to educate mothers and caregivers on HBV and available vaccinations as a means to improving the performance of HepB BD vaccination programs in Senegal (34).

Summary findings on the association between maternal education and timely HepB BD vaccination are presented in Table 5. Of the 18 studies providing information on maternal level of education, five studies found a positive relationship between educated mothers and timely receipt of the HepB BD vaccine, while five other studies found no association, and eight did not compare these two variables. Across the five studies that found significant associations between educated mothers and those without any formal education (35, 38–40, 50), the most frequent positive correlation was found between mothers with a post-secondary education and HepB BD vaccination by day 7 post-birth (35, 40, 50). These mothers were two (AOR 2.43, 95% CI [1.17–5.07]) and three times (OR 3.29, *p* = 0.02) more likely than uneducated mothers to present within 7 days for vaccination in The Gambia and Nigeria, respectively (35, 50), with one other Nigerian study observing a strong significant relationship between these variables (*p* = 0.0001) (40). Additionally, the odds of timely HepB BD vaccination by day 0–1 was higher among mothers with a post-secondary education (OR 3.6, *p* = 0.013) (35). In a Nigerian setting, mothers with a primary level of education were 17 times more likely (AOR 16.95 *p* = 0.026) to receive timely HepB BD vaccination within 24 h post-birth when compared to those with no formal education, followed by those with secondary (AOR 5.9 *p* = 0.033) and tertiary (AOR 7.7 *p* = 0.029) education (39). Health care workers in a Senegalese qualitative study believed uneducated pregnant women or mothers were less compliant with vaccination schedules (44). Overall, findings from these studies suggest that any level of formal education among pregnant women and mother may have a positive influence on the performance and outcomes of HepB BD vaccination programs in the WHO AFRO.

**Table 5:** Summary findings on the association between maternal education and timely HepB BD vaccination.

Study (Author, year)	Country	Participants (*N*)	Maternal level of education (%)				Association between education and timely vaccination	Summary findings
			No education	Primary	Secondary	Tertiary		
Okenwa et al. (2019) (34)	Nigeria	344	1.2	7.6	61.1	30.2	Not measured	37.2% of participants recommended improving HBV education of mother’s caregivers
Okenwa et al. (2020) (36)	Nigeria	366	1.2	7.6	61.1	30.2	Tertiary education was associated with valid birth-dose (COR = 1.7; AOR = 1.2)	Tertiary education was associated with good maternal knowledge of HBV infection and vaccination
Olakunde et al. (2021) (37)	Nigeria	6,143	43.1	14.3	33.4	9.3	Not measured	Level of maternal education was positively associated with receipt of vaccination when delivered at home and in public facilities
Périères et al. (2021) (53)	Senegal	241	66.5	20.1	13.4^a^	a	Level of maternal educational was not associated with non-adherence to birth-dose schedule (*p* = 0.363)	Level of maternal education was not significantly associated with non-adherence to birth-dose schedule
Sadoh et al. (2014) (41)	Nigeria	150	2.7	27.7	23.6	45.3^b^	Not measured	Overall low timely birth-dose coverage. Age, sex and socioeconomic status found not to be associated with hepatitis B seropositivity.
Ibrahim et al. (2022) (39)	Nigeria	400	3.4	8.3^c^	49.0	39.3	Education and timeliness AOR: primary = 17; secondary = 5.9; tertiary = 7.7	The level of maternal education associated with timeliness. Primary level education showed the biggest association compared to mothers with no education
Ibraheem et al. (2019) (35)	Nigeria	480	1.7^d^	^d^	34.2	64.2	Post-secondary education and presenting on day 0-1: OR = 3.6; day 2-7 OR = 3.29	Post-secondary education was significantly associated with valid timely dose of HepB BD
Danjuma et al. (2020) (42)	Nigeria	355	2.3	5.9	23.9	67.9	No significant correlations at any level (primary: *p* = 0.95; Secondary: *p* = 0.11; Tertiary: *p* = 0.65)	Level of maternal education was not significantly associated with delayed birth-dose vaccination
Bada et al. (2022) (59)	Nigeria	409	33.2^d^	^d^	66.8^a^	^a^	Level of education ≤ elementary schooling or ≥ secondary schooling was not associated with timely birth-dose (*p* = 0.63)	No association found between maternal level of education and timely birth-dose
Ibraheem et al. (2022) (38)	Nigeria	1952	6.3%	4.3%	51.2%	38.1%	Tertiary education and presentation within day 1: OR = 1.6; (*p* = 0.028)	Tertiary education was significantly associated with presentation for vaccination within day 1 post-birth
Bassoum et al. (2021) (58)	Senegal	726	57.4%	42.6%^e^	^e^	^e^	Not measured	Factors associated with co-administration of birth-dose vaccinations did not include maternal education
Bassoum et al. (2022) (57)	Senegal	832	54.1	45.9^e^	^e^	^e^	Educated vs uneducated mothers and vaccination within 24 h: *p* = 0.503	No significant association between education level and vaccination within 24 h
Dagnew et al. (2020) (60)	Ethiopia	1,121	27.5^f^	15.4	26.9	29.5	Not measured	Education was significantly associated with good HBV knowledge and attitude among pregnant women
Goodman et al. (2013) (49)	Nigeria	300	Study = 36.7; Control = 37.7	Study = 39.3 Control = 41.4	Study = 16.7 Control = 14.0	Study = 7.3 Control = 7.3	Not measured	No multivariate analysis was done
Guingané et al. (2020) (54)	Burkina Faso	2,220	35.6	21.9	37	5.5	Not measured	Interestingly a ≥ secondary level of education of both parents was significantly associated with better retention to care (more so in fathers than mothers)
Miyahara et al. (2016) (50)	The Gambia	10,851	15.8 67.1^g^	10.5	6.7	–	Higher educated mothers and vaccination by day 7 compared to uneducated mothers: AOR 2.43 (*p* = 0.02)	Vaccine coverage by day 7 was significantly higher in children born to mothers with higher levels of education
Sadoh et al. (2013) (40)	Nigeria	153	^d^	72.5^d^	27.5^a^	^a^	≥ Secondary education more likely to present within the first week of life (*p* = 0.0001)	Mothers educated beyond secondary level more likely to present for vaccination within the first week after birth
Nankya-Mutyoba et al. (2021) (48)	Uganda	70	–	48.6^h^	51.4^h^	–	Not measured	Participants were grouped by residence and education level. No other insights drawn on maternal education level

a Combined secondary and tertiary education.

b Combined university degree or equivalent (and school certificate with teaching/other professional training).

c Combined primary and Islamic education.

d Combined no education and primary education.

e Combined primary, secondary, and tertiary education.

f Combined no education and basic literacy.

g Koranic education.

h Purposive selection of education level for qualitative inquiry.

Both neonatal and maternal health concerns post-birth were reported to influence delayed HepB BD vaccination. The proportion of mothers identifying their ill health as a reason for delayed presentation for vaccination ranged from 7.6% (35), 12.3% (40) to 16.2% (38), whereas those who identified having undergone a caesarean section as their reason ranged from 5.9% (35) to 6.1% (39). In Uganda women felt they needed to recover from the stress of childbirth before their newborns could be safely vaccinated, while others who underwent an operation suggested delaying vaccination till the day of discharge (48). Superseding maternal ill health was the health and well-being of the neonates. The baby’s ill health was cited among reasons for delayed HepB BD vaccination in five studies (35, 39, 40, 42). The proportion of mothers identifying their neonates’ ill health as a deterrent to timely vaccination ranged from 5.8% (42), 9.7% (35), 10.5% (39), 11.6% (40), to 24.4% (38) across included studies. Among these studies, a lesser proportion of mothers identified prematurity as their reason for delayed vaccination (4 and 0.8% in two studies) (35, 42).

Where maternal socio-economic determinants of timely HepB BD vaccination were addressed, maternal occupation was found to be significantly associated with timely vaccination in three studies, all of which were conducted in Nigeria (38–40). In a study conducted by Ibrahim et al. (39), types of occupations were categorized into five groups, with group 1 being the higher end comprising of occupations like senior civil servants, and group 5 the lower end representing those who were students or unemployed. Group 2 (non-academic professionals like nurses, medium size business owners, secondary school teachers, intermediate grade public servants) was negatively associated with timely receipt of the HepB BD vaccine within 24 h (AOR 0.14, 95% CI [0.037-0.554]) (39). Another study found that the likelihood of vaccination by day 0–1 among petty traders and teachers was 4 and 1.5 times higher, respectively, than that among the unemployed (39). Sadoh et al. (40), applied a social class variable which combined ratings assigned for both parents’ occupation and education level, with social class 1 being the lower end and class 4 the higher end of the spectrum. High social class was found to have a statistically significant association with presentation for vaccination within the first week after birth (40).

Closely related to maternal occupation, the influence of maternal wealth on the receipt of HepB BD vaccine was reiterated among studies. In three studies assessing the relationship between these variables, women’s level of wealth was categorized in one of 5 quintiles, with the upper end being the richest and the lower end the poorest (37, 50, 58). No marked difference in the distribution of the population among the wealth quintiles were found in all three studies (37, 50, 58). Two of the three studies found no statistically significant correlation between maternal wealth and vaccination by day 7 (50), or co-administration of birth-dose vaccines (58). Contrary to this, Olakunde et al., found that wealthier mothers had higher odds of receiving HepB BD vaccination when compared to the poorest category (AOR richest =3.05, richer =2.17, middle =1.55) (37). Noteworthy were the findings on maternal unemployment despite secondary education attainment. Most mothers in one Nigerian study were unemployed (48.7%) despite the majority attaining a secondary level of education (51.2%) (38). Similar findings in Nigeria demonstrate 50% of mothers with at least a secondary level education but high unemployment (59%), additionally the majority (47.8%) belonged to the middle class (39).

Among the included studies, maternal history of ANC attendance was another determinant of timely HepB BD vaccination. Antenatal services or facilities were also frequently identified as the preferred location or medium of attaining knowledge on HBV and vaccinations, see Table 6. In Nigeria, women who attended ANC consultations were 10 times more likely to present for vaccination by day 0–1 (AOR 9.55, 95%CI [1.75–52.12]) and nearly 6 times more likely to present by day 2–7 (OR 5.78, 95%CI [1.27–26.28]) compared to those who did not attend ANC (35). Across other WHO AFRO member states, HepB BD vaccine coverage rates were shown to be high in instances of high ANC attendance (33). Similar correlations between ANC attendance and timely administration of the HepB BD vaccine were however not demonstrated in other studies (38, 39, 53). Pregnant women in Uganda preferred getting information on HBV and vaccines at their ANC consultations as opposed to via post or electronic media (43). In an exploration of the source of health information available to mothers, the health system was identified as the main source specifically on commencement of birth-dose vaccination (57.7%), of which 20.3% of mothers named ANC sessions as their source (40). Similarly, 82.2% of mothers received advice on vaccination during ANC consultations and 85.3% received advice during post-natal visits which was associated with higher odds (AOR 1.72, *p* = 0.01) of co-administration of birth-doses (58). In a related study, an increased proportion of mothers in Senegal received advice on vaccination at post-natal visits (87.2%) compared to during ANC consultations (82.4%) (57). Although paternal factors were assessed in six of the included studies, an association with HepB BD vaccination or delayed vaccine uptake was not reported (38, 39, 53, 54, 57, 58). Only the pilot program in Burkina Faso cited the level of education among fathers as being significantly associated with retention to care and HBV DNA testing among mothers, see Table 5 (54).

**Table 6:** Summary findings on ANC attendance and HepB BD vaccination.

Study (author, year)	Country	Participants (*N*)	Antenatal care attendance (%)	Summary findings
Périères et al. (2014) (53)	Senegal	241	96.2	Not attending ANC visits was not significantly associated with non-adherence to birth-dose schedule [*p* = 0.8]
Ibrahim et al. (2022) (39)	Nigeria	400	96.5	No correlation between ANC attendance and timely administration
Sadoh et al. (2013) (40)	Nigeria	153	92.2	20.3% of mothers who identified the health system as their source of information on HBV and vaccination received their information from ANC visits
Ibraheem et al. (2022) (39)	Nigeria	1952	94.7	ANC attendance was not significantly associated with vaccination at day 0-1 [p = 0.63]
Ibraheem et al. (2019) (35)	Nigeria	480	93.5	Women attending ANC were 10 times more likely to receive vaccination by day 0-1 and nearly 6 times more likely to present by days 2-7 when compared to those who did not attended ANC
Bassoum et al. (2021) (58)	Senegal	726	47.5 [<4 visits] 52.5 [≥4 visits]	82.2% of mothers received advice on vaccination during ANC visits
Bassoum et al. (2022) (57)	Senegal	832	46.4 [0-4 visits] 53.6 [≥4 visits]	82.4% of mothers received advice on vaccination during ANC visits.
*Moturi et al. (2018) (33)	Namibia	N/A	97	No comment on association between coverage and ANC attendance in these two countries
	Nigeria	N/A	61	
	Botswana	N/A	94	In these 3 countries with high coverage rates of the HepB BD (high rates of ANC attendance are recorded. ANC provides an opportunity to educate on HBV and encourage facility delivery)
	The Gambia	N/A	86	
	São Tomé and Príncipe	N/A	98	
Nankya-Mutyoba et al. (2021) (48)	Uganda	N/A	n/a	Pregnant women prefer receiving HBV education during ANC consultations

ANC, antenatal care; N/A, not applicable; *data regarding ANC attendance was derived from UNICEF 2016 report (www.data.unicef.org).

#### 3.2.8 Influence of wider contextual factors on timely HepB BD vaccination

It is critical to address how HepB BD vaccination programs perform in the local contexts where they are delivered. From our review of the evidence-base we identified key factors that influence, to varied degrees, how HepB BD vaccination programs function. These include geographical factors, cultural and religious beliefs or observances underpinning decision-making around home deliveries and post-birth practices, parental decision-making authority on a child’s health, concepts around maternal marital status and birth order of children, and the local historical or current political climate. With regards to geographical factors, physical distance, and climate issues such as seasonal weather conditions were highlighted in several studies as influencing the timeliness of HepB BD vaccination. In Nigeria for example, mothers attributed delayed HepB BD vaccination to an increased distance between their place of residence and vaccination sites, often requiring unaffordable transportation costs (34, 35, 38). Miyahara et al. (50), report that in The Gambia, increased distances of ≥2 km from the vaccination site decreased likelihood of vaccination by day 7 (AOR 0.41 [*p* < 0.0001]) but those residing in rural areas were more likely to be vaccinated by day 7 compared to those from urban or peri-urban areas (West rural AOR 6.13; East rural AOR 6.72 [*p* < 0.001]). Even when assessing correlation of vaccination by day 1, rural areas faired significantly better than urban or peri-urban areas (AOR 4.61, 95% CI [2.27-9.36]) in this study (50). Two health system design factors were advantageous to this Gambian cohort; 50% of infants lived within 1 km of vaccination clinics and village HCWs performed an active role in informing rural mothers of the dates of outreach clinics (50). Unlike their counterparts in The Gambia, pregnant women residing in urban areas in Ethiopia were two times more likely than rural residents to have good attitudes towards HBV transmission, screening, and vaccination (60). By adopting a service delivery structure involving three strategies, fixed, advanced, and mobile strategies, Senegal has been able to expand access to vaccination services (57, 58). The fixed strategy is designed to provide vaccination services at fixed health centers to those living within a 5 km radius, while the advanced strategy targets those staying between 5–15 km from health centers with services rendered at health huts or sites by the staff from the main health centers. The mobile strategy on the other hand, targets those living >15 km from the health centers (57). With this service model, Bassoum et al. (58), observed that 66.1% of mothers lived within 5 km from a health center and this was found to be an enabling factor for co-administration of birth-dose vaccines. Interestingly, in another study by Bassoum et al. (57), findings showed that although 70.1% of the sample population lived <5 km from a health center, this was not associated with HepB BD vaccination within 24 h of life. In addition to physical distance, it has been demonstrated that being born in the dry season is associated with a 1.97 times higher likelihood of non-adherence to the HepB BD vaccination schedule when compared to those born in the wet season (53). Reasons proffered in a Senegalese study for this outcome include migration during the dry seasons which reduced the likelihood of adherence to the vaccination schedule (53).

With regards to home birthing practices, a survey conducted among African experts found that 92% reported limited vaccine resources for neonates born outside of health facilities (47). In this same study, ~22% of participating African countries reported that the proportion of deliveries outside of health facilities was in the region of 40% or higher (47). In a Nigerian study where the majority of participants were rural residents (60.5%), over 50% of women delivered at home with a high rate of unskilled birth attendants (54.1%) (37). Thirty-three percent of those who delivered at home received HepB BD vaccination whereas the vaccine coverage rate among neonates delivered in both private and public health facilities was over 75% (37). It is also worth noting that of those who did not receive their HepB BD vaccine, majority (69.5%) were delivered by unskilled birth attendants (37). Home deliveries in Senegal were also associated with non-adherence to the HepB BD vaccination schedule (AOR 2.02, *p* = 0.07) (53). In The Gambia, home deliveries (40.3%) and assistance by traditional birth attendants or TBAs (29.8%) are prominent components of the broader health system (50). In this primary health care system, village based TBAs and village HCWs are supervised by community nurses (50). Contrary to findings from Nigeria and Senegal, timely vaccination in The Gambia has been shown to favor those infants born at home. While coverage remains unacceptably low, relatively higher vaccine uptake by day 0–1 for home deliveries (1.3%) compared to deliveries in health centers (0.8%) and hospitals (0.5%) likely reflect the health systems design in The Gambia which accommodates the local realities of home deliveries (50). There is clear demand for designing vaccination systems that make careful considerations for long-established birthing practices rather than dismantling them altogether.

While we anticipated that ethical norms, cultural practices, and religion would be important considerations for timely uptake of the HepB BD vaccine, such topics were rarely addressed in the evidence-base. Some of the limited data available highlighted how mothers from some core northern states in Nigeria were discouraged from leaving their homes with their babies before the name giving ceremony held on day 7 post-birth (38). Accordingly, it was reported that 6.4% of mothers delayed vaccination until after the naming ceremony (38) as did those participating in another Nigerian study where 6.5% of the mothers delayed presenting for vaccination as they were “waiting for after the naming ceremony” (39). This was also highlighted as a cultural practice in both Nigeria and The Gambia in the multi-country study (33). Additionally, waiting to circumcise male babies seven days after birth was given as a reason by 3.2% of mothers in a study conducted in Nigeria (40). In Uganda, a study investigating maternal perceptions and preferences of HepB BD vaccination highlighted participants’ belief that newborns should not be out of the mothers’ sight in order to remain protected. Mothers suggested the handling of newborns be done in their presence, especially during vaccination (48). Another cultural perspective cited in two of the included studies was the decision-making authority within the household. Only 0.6 and 1.1% of mothers participating in the two studies proffered paternal non-consent as a reason for delayed presentation for HepB BD vaccination (38, 39). Another study identified the unavailability of husbands among reasons for delayed presentation for HepB BD vaccination (40). In Senegal, one study found that 97.5% of decisions concerning the child’s health were made by the mother, or both the mother and father, as opposed to somebody other than the parent (58).

Findings from a long-term observational study in The Gambia noted participants from the Fula ethnicity had significantly lower odds (AOR 0.60, 95%CI [0.40-0.91]) of receiving the HepB BD vaccine by day 7 compared to the majority Wollof ethnicity (50). In Senegal, among the Serer ethnic population, HBV was likened to a dietary problem commonly managed by traditional medicine (44). In Ethiopia, HBV is known as “Yewefe Bashita” and thought to be transmitted through bat feces and urine, and as such, the local population was unaware of the importance or need for clinical treatment or prevention (60). Religion as a contextual determinant was assessed in three studies, two of which found a significant association with HepB BD vaccination. Infants born to Christian mothers in Nigeria had twice the odds of vaccination by day 0–1 than those born to Muslim mothers (38). Another Nigerian study found that religion was a significant determinant for home births (37), where the odds of receiving HepB BD vaccination were 0.66 times lower among Muslims when compared to Christians (37). A noteworthy related finding is the fact that 61.3% of the population in the latter study prescribed to the Islamic faith (37).

In terms of birth order, findings appeared inconclusive among three Nigerian studies reporting on the determinant of timely vaccination (38, 39, 59). One study demonstrated higher birth order (3rd born) increased the likelihood of HepB BD vaccination within 24 h by 6-fold when compared to the first born (39). To the contrary, lower birth order (between 2nd–4th born) was associated with 1.5 times the odds of timely vaccination when compared to the 5th born in another study (39), whereas no association between parity and timely vaccination was found in the other study (59). In Loarec et al. (52), authors discuss concerns of the high fertility rate in Mozambique (4.85 births per female in 2018) which when considered together with high home birth rates in some non-urban settings has important implications for access to timely vaccination. Globally, the increasing number of live births per woman was found to be inversely proportional to HepB BD vaccination coverage (*p* = 0.01) (51). In this regard, discussions in this publication centered around higher birth rates likely overwhelming the health system and thereby impacting the capacity to provide timely birth-dose vaccines (51). Lastly, only two studies addressed the influence of conflicts or unrest on the performance of HepB BD vaccination programs. In examining the low coverage and timeliness of HepB BD vaccination in 2018 compared to that in 2017 in Senegal, Périères et al. (53), found that a HCW strike which took place between April–December 2018 had a considerable effect on national immunization services. Aina et al. (43), on the other hand, highlighted the insecurities across the north-eastern parts of Nigeria which caused migrations to stable states like Kano in the north-western parts of the country, and thereby negatively impacting on timely uptake of HepB BD vaccination.

## 4 Discussion

With 2030 drawing close, more countries within the WHO AFRO plan to introduce selective or universal hepatitis B birth dose vaccination programs as part of national viral hepatitis elimination strategies (4). We contribute synthesized evidence on the complexities influencing the performance of hepatitis B birth dose vaccination programs with the aim of informing the strengthening of future and existing programs in the region.

Where the intervention itself is concerned, the source of complexity lies with the permitted degree of flexibility in the timely administration of the HepB BD vaccine for optimal PMTCT of HBV (17). This further interacts with complexities prevalent across the causal pathway of the vaccination program. This is demonstrated in the dynamics involved in the translation of policy or guidelines into practice (17). We found impaired feedback loops created by misinterpretation of policy encouraged multiple stakeholders to continue a pattern of non-adherence to timely vaccination. When national policies allow for a 0–14-day timeframe for the receipt of the HepB BD vaccine (33–37), HCWs are likely to interpret the upper limit as inferring the same protection as a dose received within 24 h. This misinterpretation would impact on their decisions and practices which in turn influences the health seeking behaviors of mothers leading to a cascade of delayed behaviors. Consequently, mothers presenting for vaccination within 14 days was the most frequent timeframe noted in this review (38–40). Policy makers should take care to not compromise effective program performance when adapting international guidelines to local contexts. Similar complexities have been noted with birth-dose co-administration practices serving convenience or wastage aversions in some settings (50). Reasons for delaying HepB BD vaccination while awaiting pairing with OPV or BCG (39, 50) need to be further investigated in order to formulate pragmatic solutions that do not compromise vaccine effectiveness. Although our findings demonstrate that deficits in supply are not the sole reason for poor program performance, it remains as an important source of complexity (34, 38–40, 42, 48). This has also been demonstrated in other reviews on the performance of HepB BD vaccination programs (6, 14, 15). Establishing a sustainable supply of the HepB BD vaccine decreases the likelihood of untimely or missed vaccination (6). It might be that more innovative strategies, like direct-to-facility supply, could avoid bottlenecks and improve effective program performance (43).

The design of the intervention was also observed as an important point of complexity. Most infant vaccination programs are delivered on allotted days at immunization centers or clinics (35, 38, 39, 42, 59, 61). Though this has allowed for the delivery of essential vaccines as part of the EPI globally (62, 63), this design feature is not the best fit for HepB BD vaccination programs as it leads to poorly accessible services. This is further compounded by several influential maternal and wider contextual factors such as maternal knowledge and awareness of the risk and prevention of HBV MTCT (35, 36, 40), health status of mothers and infants post-birth (35, 39, 40, 42), cultural and religious practices (33, 37–39), geographical factors and seasonal changes (34, 35, 38), home birthing preferences (37, 50, 57) and maternal occupation and level of education (35, 38–41). These characteristics act as mediators or moderators of the intervention (17). Aligning HepB BD vaccination with birth delivery services would be an important step in overcoming this complexity, allowing for a more responsive intervention design that encourages effective vaccination practices. Such efforts should include, pre-positioning of vaccines in delivery rooms (34, 46); ordering of single dose vials or compact pre-filled auto-disable injections (CPADs) for use in delivery centers and during home births (64), the use of mobile vaccination initiatives combined with the use of the vaccine outside the cold-chain (65), training TBAs or village HCWs on the use of CPADs for countries with high volumes of home births (64), and formulating policies that shift responsibility of vaccine administration to the birthing facility or agent as opposed to immunization centers (65, 66). These strategies have proven useful in other settings with similar contexts (64–66).

Further to changes aimed at the design of the intervention, changes in the moderators of effect, like maternal and contextual factors, could provide systemic change in the performance of the vaccination program. Where cultural or religious practices such as naming ceremonies and male circumcision influence delayed uptake of the HepB BD vaccine, explorations of these socio-cultural practices should be conducted and carefully accommodated as part of the vaccination program in order to establish trust from local communities. This calls for strategic planning and social mobilization, engaging community, cultural, and religious leaders to negate misconceptions, raise awareness and improve acceptance of the vaccination program. These cultural considerations are not unique to the WHO AFRO. A previous study suggests that mothers in Indonesia are encouraged to remain indoors with their newborns during the first 40 days of life (64). In this study it was reported that health promotion activities like face-to-face educational sessions during ANC visits, health promotion material such as handouts and mass media campaigns via radio communication improved acceptance of the vaccination program among local communities in Indonesia (64). Further to this, our review noted the pivotal role of village HCWs and TBAs who are essential in raising awareness on outreach immunization services and improving timely uptake of HepB BD vaccine in rural settings with substantial home birthing practices (50). Similar strategies have been used in Papua New Guinea where village HCWs are critical to raising awareness (67).

When considering the broader health system, sources of financial resources described as contributing to vaccine coverage include government health spending, donor funding or development assistance for health, out-of-pocket and prepaid private health spending (68). Among low-income countries, an increase in total health expenditure does not always translate into better health outcomes or optimal vaccine coverage (68, 69). In contrast, national or government health spending *per capita* and government spending per birth on routine vaccines, have been proven as positive predictors of vaccination coverage (68, 69). A steady increase in national funding for new vaccine introductions, like the HepB BD vaccine, in the WHO AFRO is likely to improve coverage. This review highlights how inadequately resourced HepB BD vaccination programs can result in exorbitant out-of-pocket payments which are important constraints to end-user buy-in and uptake of services. In addition, these findings give impetus to the ongoing calls for relevant stakeholders, including global partners like Gavi, to further their pivotal role across the region and honor their financial commitments to support the strengthening of existing programs while expanding roll-out of nationwide HepB BD vaccination programs across the region (70, 71). In 2018, as part of their investment strategy, Gavi committed to providing support for HepB BD vaccination by 2021 but due to the COVID-19 pandemic, these intentions were deferred, although currently being reconsidered following an impressive global movement (70). Even with Gavi support, it is imperative that national governments mobilize domestic investments as this has been shown to strengthen country ownership and secure the sustainability of the vaccination program above dependence on donor funding (68). The China-Gavi project is an example of one such collaboration that helped to convince the Chinese government to introduce and fully fund HepB BD vaccination after attaining 75% coverage in 80% of Gavi project counties (72).

We also found that the level of HBV specific knowledge among HCWs created behavioral change in end-users and HCWs themselves. The poor level of HBV specific knowledge among HCWs manifested in delayed vaccination, lenient practices when screening for selective vaccination, and inaccurate or poor knowledge transfer from providers to mothers and pregnant women (45, 48, 52, 56). This emphasizes the importance of increasing the basic knowledge among all HCWs, especially those involved with MCH activities as they are the first point of contact for pregnant women and the preferred source of HBV-related information (48, 58). Improving the level of knowledge about HBV among HCWs is likely more feasible when training is integrated with other disease training models, like that of HIV (73). This serves as a low-cost intervention towards HBV elimination (74). Future research directions should include exploring potential gaps in tertiary or formal training of HCWs in order to advise the Ministry of Education in adapting the curriculum to local contexts. Dedicated educational sessions and training on HBV among HCWs in Tanzania and Uganda have seen improvements in HBV knowledge but call for ongoing efforts to sustain improved basic knowledge among HCWs (73, 74). Elloker et al. (75), highlight the importance of embracing the ‘tangible software’ like knowledge, skills, systems and procedures, as well as the “intangible software” such as values, norms, power, communication, and relationships. In our review, knowledge and awareness (tangible software) among HCWs were investigated more frequently than their values, norms, communication, or relationships (intangible software). However, we found dynamics of trust and power (intangible software) evident between HCWs and mothers in the handling of newborns and administration of vaccines (48, 49). It would be premature to draw conclusions on this potentially rich source of complexity based on our limited findings given the gap in research. Further research is needed to better explore these dynamics and how they influence the performance HepB BD vaccination programs.

## 5 Strengths and limitations of this review

To the best of our knowledge, this qualitative systematic review is the first to explore how key underlying complexities influence the performance of HepB BD vaccination programs in the African region. We retrieved and critically appraised literature sources published in both English and French and indexed in multiple electronic databases and repositories. By applying a systems-based logic model developed in a preceding scoping exercise and tailored to systematic reviews of complexity, we enhanced the reliability and validity of our data collection, synthesis, and analysis. Limitations in the generalizability of the review findings lie in the underrepresentation of other WHO AFRO member states while studies from countries like Nigeria and Senegal dominated the knowledgebase. However, it is important to consider that only 15 member states have so far adopted national HepB BD vaccination policies. In addition, research capabilities and appetites may vary even across those same countries. Systematic review designs are subject to the biases and confounders inherent in component studies, and this should be considered when interpreting the findings of this review.

## 6 Conclusion

This systematic review draws on the complex links between the design of hepatitis B birth dose vaccination programs and the broader health systems that deliver them, providing complex explanations as to why simply introducing the vaccine may not lead to timely uptake or improved coverage. Owing to the complexity of the hepatitis B birth dose vaccination program, or the complex interaction with the health system, findings and recommendations on strengthening program performance are expected to be multifaceted. Our findings underscore five major considerations for scaling up HepB BD vaccinations in the WHO AFRO. Firstly, the misinterpretation of policy significantly contributed to poor program performance. This produces a cascade of adaptations and behavioral changes along the chain of relevant stakeholders which negatively influences timely vaccine uptake and may ultimately derail HBV PMTCT efforts. Research exploring the non-adherence to policy guidelines is largely lacking despite its systemic effect on implementation and control of HBV in Africa. We therefore encourage further investigation of this focused topic in order to inform interventions that enhance HCW adherence and maximize the benefits of HepB BD in the region. Secondly, the existing design of the program including information systems and supply chains may be inadequate in meeting the needs of an intervention with complex requirements like the HepB BD vaccination program. Innovative and context-specific approaches are required in order to ensure programmatic success. Thirdly, acknowledging the contextual underpinnings and multiple influencing factors of end-users is pertinent when designing and implementing this program. Fourthly, recognizing the role of various cadres of HCWs as a reliable source of information, vaccine administrators, and as complex individuals themselves, is essential to providing tailored support and improving the delivery of the program. Lastly, national governments’ buy-in in mobilizing financial resources and maintaining intersectoral collaboration among MoH, education and social development would provide a sustainable basis for programmatic success within the region.

Ultimately, countries within the WHO AFRO looking to introduce, or scale-up HepB BD vaccination programs will benefit from carefully considering components of the intervention design that require responsiveness and flexibility (vaccine accessibility and delivery), or inflexibility (policy interpretation); which stakeholders require further support (HCWs and government ministries); and where innovation is required (information systems and supply chains). Lessons learned from the experiences of the various African countries clearly demonstrate that successful introduction and implementation of HepB BD vaccination programs across the region is achievable with careful consideration of complexities within the broader health system.

## Data Availability

The original contributions presented in the study are included in the article/Supplementary material, further inquiries can be directed to the corresponding author.
