# Supplementary material for: Towards contextualized complex systems approaches to scaling-up hepatitis B birth-dose vaccination in the African region: a qualitative systematic review
Source: Front Public Health. 2024 Oct 24;12:1389633. doi: 10.3389/fpubh.2024.1389633 (PMC11540787; doi:10.3389/fpubh.2024.1389633)
Supplement: Supplementary file 2 [file Data_Sheet_2.PDF]

Supplementary File 2. Literature search strategy per databases

PubMed Database	
**Search**	**Query**
#1	(Infant[MeSH Terms]) OR (child[Title/Abstract] OR children[Title/Abstract] OR infant[Title/Abstract] OR infants[Title/Abstract] OR neonate[Title/Abstract] OR neonates[Title/Abstract] OR newborn[Title/Abstract])
#2	(Hepatitis B OR Hepatitis B vaccines[MeSH Terms]) OR (Hepatitis B[Title/Abstract] OR hep B[Title/Abstract] OR HBV[Title/Abstract] OR hepatitis B birth dose[Title/Abstract] OR hepatitis B birth-dose[Title/Abstract])
#3	(Immunization[MeSH Terms]) OR (immunization[Title/Abstract] OR immunisation[Title/Abstract] OR immunization coverage[Title/Abstract] OR immunisation coverage[Title/Abstract] OR immunisation programs[Title/Abstract] OR immunization programs[Title/Abstract] OR immunization programmes[Title/Abstract] OR immunisation programmes[Title/Abstract] OR immunization initiatives[Title/Abstract] OR immunisation initiatives[Title/Abstract] OR Vaccine[Title/Abstract] OR vaccines[Title/Abstract] OR vaccination[Title/Abstract] OR vaccine coverage[Title/Abstract])
#4	#2 AND #3
**#5**	Healthcare workers[Text Word] OR healthcare professionals[Text Word] OR traditional birth attendants[Text Word] OR midwife[Text Word] OR midwives[Text Word] OR community health workers[Text Word] OR voluntary worker[Text Word] OR training[Text Word] OR skills building[Text Word] OR staff responsibilities[Text Word] OR standards[Text Word] OR staffing[Text Word] OR human resource[Text Word] OR human resources[Text Word] OR task shifting[Text Word] OR home care service[Text Word] OR community care[Text Word] OR outreach[Text Word] OR home visit[Text Word] OR cold chain[Text Word] OR cold temperature[Text Word] OR supply chain[Text Word] OR distribution[Text Word] OR drug storage[Text Word] OR medicine storage[Text Word] OR vaccine storage[Text Word] OR Uniject[Text Word] OR compact pre-filled auto-disabled device[Text Word] OR CPAD[Text Word] OR vials[Text Word] OR dose[Text Word] OR dosage[Text Word] OR birth delivery[Text Word] OR birth centre[Text Word] OR birth center[Text Word] OR health centre[Text Word] OR health center[Text Word] OR health facility[Text Word] OR health facilities[Text Word] OR monitor[Text Word] OR monitoring[Text Word] OR register[Text Word] OR registers[Text Word] OR registration[Text Word] OR record[Text Word] OR data collection[Text Word] OR organization[Text Word] OR organisation[Text Word] OR administration[Text Word] OR management[Text Word] OR integrated[Text Word] OR immunisation program[Text Word] OR immunization program[Text Word] OR immunisation programs[Text Word] OR immunization programs[Text Word] OR immunisation programme[Text Word] OR immunization programmes[Text Word] AND cost analysis[Text Word] OR costs[Text Word] OR funds[Text Word] OR funding[Text Word] OR finance[Text Word] OR finances[Text Word] OR financial resource[Text Word] OR financial resources[Text Word] OR Immunisation program standards[Text Word] OR Immunization program standards[Text Word] OR immunisation programme standards[Text Word] OR immunization programme standards[Text Word] OR immunisation schedule[Text Word] OR immunization schedule[Text Word] OR timeliness[Text Word] OR timely[Text Word] OR 24 hours[Text Word] OR 24-hours[Text Word] OR knowledge[Text Word] OR attitudes[Text Word] OR practice[Text Word] OR practices[Text Word] OR Policy makers[Text Word] OR policymakers[Text Word] OR policy[Text Word] OR policies[Text Word]
#6	(Public Health Systems Research OR Delivery of Healthcare[MeSH Terms]) OR (Barriers[Text Word] OR constraints[Text Word] OR obstacles[Text Word] OR challenges[Text Word] OR facilitators[Text Word] OR enablers[Text Word] OR factors[Text Word] OR determinants[Text Word] OR geographical barriers[Text Word] OR Seasons[Text Word] OR rainy season[Text Word] OR terrain[Text Word] OR distance[Text Word] OR access[Text Word] OR accessibility[Text Word] OR service access[Text Word] OR service accessibility[Text Word] OR quality[Text Word] OR availability[Text Word] OR affordability[Text Word] OR timing[Text Word] OR health decision making[Text Word] OR health decision-making[Text Word] OR maternal decision-making[Text Word] OR maternal decision making[Text Word] OR maternal autonomy[Text Word] OR maternal education[Text Word] OR maternal experience[Text Word] OR postnatal practices[Text Word] OR post natal practices[Text Word] OR post-natal practices[Text Word] OR socio-cultural practices[Text Word] OR cultural practices[Text Word] OR traditional beliefs[Text Word] OR Health systems[Text Word] OR Healthcare systems[Text Word] OR health systems performance[Text Word])
#7	#5 OR #6

#8	(Africa[MeSH Terms]) OR (Africa[Text Word] OR African[Text Word] OR Algeria[Text Word] OR Angola[Text Word] OR Benin[Text Word] OR Botswana[Text Word] OR "Burkina Faso"[Text Word] OR Burundi[Text Word] OR “Cabo Verde”[Text Word] OR Cameroon[Text Word] OR Cameroun[Text Word] OR "Canary Islands"[Text Word] OR "Cape Verde"[Text Word] OR "Central African Republic"[Text Word] OR Chad[Text Word] OR Comoros[Text Word] OR Congo[Text Word] OR "Cote d'Ivoire"[Text Word] OR "Democratic Republic of Congo"[Text Word] OR Djibouti[Text Word] OR Egypt[Text Word] OR Eritrea[Text Word] OR eSwatini[Text Word] OR Ethiopia[Text Word] OR Gabon[Text Word] OR Gambia[Text Word] OR Ghana[Text Word] OR Guinea[Text Word] OR Guinea- Bissau[Text Word] OR "Ivory Coast"[Text Word] OR Jamahiriya[Text Word] OR Kenya[Text Word] OR Lesotho[Text Word] OR Liberia[Text Word] OR Libya[Text Word] OR Madagascar[Text Word] OR Malawi[Text Word] OR Mali[Text Word] OR Mauritania[Text Word] OR Mauritius[Text Word] OR Mayotte[Text Word] OR Morocco[Text Word] OR Mozambique[Text Word] OR Namibia[Text Word] OR Niger[Text Word] OR Nigeria[Text Word] OR Principe[Text Word] OR Reunion[Text Word] OR Rwanda[Text Word] OR “Saint Helena”[Text Word] OR “Sao Tome”[Text Word] OR Senegal[Text Word] OR Seychelles[Text Word] OR “Sierra Leone”[Text Word] OR Somalia[Text Word] OR “St Helena”[Text Word] OR Sudan[Text Word] OR Swaziland[Text Word] OR Tanzania[Text Word] OR Togo[Text Word] OR Tunisia[Text Word] OR Uganda[Text Word] OR “Western Sahara”[Text Word] OR Zaire[Text Word] OR Zambia[Text Word] OR Zimbabwe[Text Word])
#9	#1 AND #4 AND #7 AND 8
#10	**Filters: Abstract availability, Full Text availability, Human studies, Published date 2009 – 2022**
**EBSCOhost Database**	
**Search**	**Query**
#1	AB Child* OR infant* OR neonat* OR newborn*
#2	AB (Hepatitis B OR hep B OR HBV OR hepatitis B birth dose OR hepatitis B birth-dose) AND AB (Immuni* OR immuni* coverage OR immuni* program* OR immuni* initiatives OR Vaccin* OR vaccin* coverage)
#3	AB (Healthcare workers OR healthcare professionals OR traditional birth attendants OR midwife OR midwives OR community health workers OR voluntary worker OR training OR skills building OR staff responsibilities OR standards OR staffing OR human resource* OR task shifting OR home care service OR community care OR outreach OR home visit OR cold chain OR cold temperature OR supply chain OR distribution OR drug storage OR medicine storage OR vaccine storage OR Uniject OR compact pre-filled auto-disabled device OR CPAD OR vials OR dose OR dosage OR birth delivery OR birth centre OR birth center OR health centre OR health center OR health facility OR health facilities OR monitor* OR regis* OR record OR data collection OR organization OR organisation OR administration OR management OR integrated OR immuni* program* AND cost analysis OR costs OR funds OR funding OR finance OR finances OR financial resource* OR Immuni* program* standards OR immuni* schedule OR timeliness OR timely OR 24 hours OR 24-hours OR knowledge OR attitudes OR practice* OR Policy makers OR policymakers OR polic*) OR AB (Barriers OR constraints OR obstacles OR challenges OR facilitators OR enablers OR factors OR determinants OR geographical barriers OR Seasons OR rainy season OR terrain OR distance OR access OR accessibility OR service access OR service accessibility OR quality OR availability OR affordability OR timing OR health decision making OR health decision-making OR maternal decision-making OR maternal decision making OR maternal autonomy OR maternal education OR maternal experience OR postnatal practices OR post natal practice* OR post-natal practice* OR socio-cultural practice* OR traditional beliefs OR Health systems OR Healthcare systems OR health systems performance)
#4	AB Africa OR African OR Algeria OR Angola OR Benin OR Botswana OR "Burkina Faso" OR Burundi OR “Cabo Verde” OR Cameroon OR Cameroun OR "Canary Islands" OR "Cape Verde" OR "Central African Republic" OR Chad OR Comoros OR Congo OR "Cote d'Ivoire" OR "Democratic Republic of Congo" OR Djibouti OR Egypt OR Eritrea OR eSwatini OR Ethiopia OR Gabon OR Gambia OR Ghana OR Guinea OR Guinea- Bissau OR "Ivory Coast" OR Jamahiriya OR Kenya OR Lesotho OR Liberia OR Libya OR Madagascar OR Malawi OR Mali OR Mauritania OR Mauritius OR Mayotte OR Morocco OR Mozambique OR Namibia OR Niger OR Nigeria OR Principe OR Reunion OR Rwanda OR “Saint Helena” OR “Sao Tome” OR Senegal OR Seychelles OR “Sierra Leone” OR Somalia OR “St Helena” OR Sudan OR Swaziland OR Tanzania OR Togo OR Tunisia OR Uganda OR “Western Sahara” OR Zaire OR Zambia OR Zimbabwe
#5	#1 AND #2 AND #3 AND #4
#6	**Filters: Full text availability, Published date 2009 – 2022**
Notes	Search in: Academic Search Premier; Africa-Wide Information; CINAHL; Health Source: Nursing/Academic Edition; APA PsycInfo
**Web of Science Database**	
**Search**	**Query**
#1	AB=(Child* OR infant* OR neonat* OR newborn*)

#2	(AB=(Hepatitis B OR hep B OR HBV OR hepatitis B birth dose OR hepatitis B birth-dose)) AND AB=(Immuni* OR immuni* coverage OR immuni* program* OR immuni* initiatives OR Vaccin* OR vaccin* coverage)
#3	(AB=(Healthcare workers OR healthcare professionals OR traditional birth attendants OR midwife OR midwives OR community health workers OR voluntary worker OR training OR skills building OR staff responsibilities OR standards OR staffing OR human resource* OR task shifting OR home care service OR community care OR outreach OR home visit OR cold chain OR cold temperature OR supply chain OR distribution OR drug storage OR medicine storage OR vaccine storage OR Uniject OR compact pre-filled auto-disabled device OR CPAD OR vials OR dose OR dosage OR birth delivery OR birth centre OR birth center OR health centre OR health center OR health facility OR health facilities OR monitor* OR regis* OR record OR data collection OR organization OR organisation OR administration OR management OR integrated OR immuni* program* AND cost analysis OR costs OR funds OR funding OR finance OR finances OR financial resource* OR Immuni* program* standards OR immuni* schedule OR timeliness OR timely OR 24 hours OR 24-hours OR knowledge OR attitudes OR practice* OR Policy makers OR policymakers OR polic*)) OR AB=(Barriers OR constraints OR obstacles OR challenges OR facilitators OR enablers OR factors OR determinants OR geographical barriers OR Seasons OR rainy season OR terrain OR distance OR access OR accessibility OR service access OR service accessibility OR quality OR availability OR affordability OR timing OR health decision making OR health decision-making OR maternal decision-making OR maternal decision making OR maternal autonomy OR maternal education OR maternal experience OR postnatal practices OR post natal practice* OR post-natal practice* OR socio-cultural practice* OR traditional beliefs OR Health systems OR Healthcare systems OR health systems performance)
#4	AB=(Africa OR African OR Algeria OR Angola OR Benin OR Botswana OR "Burkina Faso" OR Burundi OR “Cabo Verde” OR Cameroon OR Cameroun OR "Canary Islands" OR "Cape Verde" OR "Central African Republic" OR Chad OR Comoros OR Congo OR "Cote d'Ivoire" OR "Democratic Republic of Congo" OR Djibouti OR Egypt OR Eritrea OR eSwatini OR Ethiopia OR Gabon OR Gambia OR Ghana OR Guinea OR Guinea- Bissau OR "Ivory Coast" OR Jamahiriya OR Kenya OR Lesotho OR Liberia OR Libya OR Madagascar OR Malawi OR Mali OR Mauritania OR Mauritius OR Mayotte OR Morocco OR Mozambique OR Namibia OR Niger OR Nigeria OR Principe OR Reunion OR Rwanda OR “Saint Helena” OR “Sao Tome” OR Senegal OR Seychelles OR “Sierra Leone” OR Somalia OR “St Helena” OR Sudan OR Swaziland OR Tanzania OR Togo OR Tunisia OR Uganda OR “Western Sahara” OR Zaire OR Zambia OR Zimbabwe)
#5	#1 AND #2 AND #3 AND #4
#6	**Filters: Published date 2009 – 2022**
**Scopus Database**	
**Search**	**Query**
#1	TITLE-ABS(Child* OR infant* OR neonat* OR newborn*)
#2	TITLE-ABS(“Hepatitis B” OR “hep B” OR HBV OR “hepatitis B birth dose” OR “hepatitis B birth-dose”) AND TITLE-ABS(Immuni* OR “immuni* coverage” OR “immuni* program*” OR “immuni* initiatives” OR Vaccin* OR “vaccin* coverage”)
#3	TITLE-ABS-KEY(“Healthcare workers” OR “healthcare professionals” OR “traditional birth attendants” OR midwife OR midwives OR “community health workers” OR “voluntary worker” OR training OR “skills building” OR “staff responsibilities” OR standards OR staffing OR “human resource*” OR “task shifting” OR “home care service” OR “community care” OR outreach OR “home visit” OR “cold chain” OR “cold temperature” OR “supply chain” OR distribution OR “drug storage” OR “medicine storage” OR “vaccine storage” OR Uniject OR “compact pre-filled auto-disabled device” OR CPAD OR vials OR dose OR dosage OR “birth delivery” OR “birth centre” OR “birth center” OR “health centre” OR “health center” OR “health facility” OR “health facilities” OR monitor* OR regis* OR record OR “data collection” OR organization OR organisation OR administration OR management OR integrated OR “immuni* program*” AND “cost analysis” OR costs OR funds OR funding OR finance OR finances OR “financial resource*” OR “Immuni* program* standards” OR “immuni* schedule” OR timeliness OR timely OR 24 hours OR 24-hours OR knowledge OR attitudes OR practice* OR “Policy makers” OR policymakers OR polic*) OR TITLE-ABS-KEY(Barriers OR constraints OR obstacles OR challenges OR facilitators OR enablers OR factors OR determinants OR “geographical barriers” OR Seasons OR “rainy season” OR terrain OR distance OR access OR accessibility OR service access OR “service accessibility” OR quality OR availability OR affordability OR timing OR health decision making OR “health decision-making” OR “maternal decision-making” OR “maternal decision making” OR “maternal autonomy” OR “maternal education” OR “maternal experience” OR “postnatal practice*” OR “post natal practice*” OR “post-natal practice*” OR “socio-cultural practice*” OR “traditional beliefs” OR “Health systems” OR “Healthcare systems” OR “health systems performance”)

#4	TITLE-ABS-KEY(Africa OR African OR Algeria OR Angola OR Benin OR Botswana OR "Burkina Faso" OR Burundi OR "Cabo Verde" OR Cameroon OR Cameroun OR "Canary Islands" OR "Cape Verde" OR "Central African Republic" OR Chad OR Comoros OR Congo OR "Cote d'Ivoire" OR "Democratic Republic of Congo" OR Djibouti OR Egypt OR Eritrea OR eSwatini OR Ethiopia OR Gabon OR Gambia OR Ghana OR Guinea OR Guinea- Bissau OR "Ivory Coast" OR Jamahiriya OR Kenya OR Lesotho OR Liberia OR Libya OR Madagascar OR Malawi OR Mali OR Mauritania OR Mauritius OR Mayotte OR Morocco OR Mozambique OR Namibia OR Niger OR Nigeria OR Principe OR Reunion OR Rwanda OR "Saint Helena" OR "Sao Tome" OR Senegal OR Seychelles OR "Sierra Leone" OR Somalia OR "St Helena" OR Sudan OR Swaziland OR Tanzania OR Togo OR Tunisia OR Uganda OR "Western Sahara" OR Zaire OR Zambia OR Zimbabwe)
#5	#1 AND #2 AND #3 AND #4
#6	**Filters: Published date 2009 – 2022**
