# Supplementary material for: Towards contextualized complex systems approaches to scaling-up hepatitis B birth-dose vaccination in the African region: a qualitative systematic review
Source: Front Public Health. 2024 Oct 24;12:1389633. doi: 10.3389/fpubh.2024.1389633 (PMC11540787; doi:10.3389/fpubh.2024.1389633)
Supplement: Supplementary file 3 [file Data_Sheet_3.PDF]

Supplementary File 3. Template of the data extraction sheet

Study ID					Study/literature details											Intervention theory and design		
No.	First Author	Year	Title	Country	Type of literature	Language	Aim of study/ literature	Study design	Study duration	Participant Type	Population Size	Sampling	Data collection method	Study limitations	Ethical considerations/ Author reflexivity/ Informed consent	Practice in study: Universal or selective hepatitis B birth-dose vaccination	Presence of national guideline/policy instated on hepatitis B birth-dose vaccination	Presence of institutional guidelines/policy on hepatitis B birth-dose vaccination
1.																		
2.																		
3.																		

Intervention theory and design (continued…)					Intervention execution and delivery									
Training of delivery agents	Education/knowledge of pregnant women or mothers	Monitoring system in place: evidence of feedback and feedforward mechanisms present	Access to infrastructure/ health facility / immunization service	Access/ availability of required technologies (cold chain, vaccine)	Timing of vaccine administration	Time range allowed for vaccine administration	Adherence to data capturing	Delivered agent	Influential delivery agent characteristic/s (soft and hard skills)	Setting of delivery	Type of Vaccine used (Brand/supplier)	Mechanism of vaccine<br><br>(10 dose/single dose/auto-disabled device)	Cost of vaccine	Logistics of supply

Implementation							Context					
Policy			Financing									
Knowledge translation of policy (Top down)	Adherence to policy	Responsiveness of policy	Source of finance for intervention	Duration of finances for intervention (e.g., if donor funding)	Competing financial priorities within context of country	Organization/structural level responsible for immunization budget and delivery	Geographical barriers/ enablers	Epidemiology of the country<br><br>(Burden of hepatitis or other diseases)	Socio-economic and cultural factors<br><br>(e.g., household autonomy, societal hierarchy)	Socio-economic country climate	Ethical norms	Political structure (e.g., unrest/ monarchy/ democracy/corruption)

Outcomes				
**Process outcomes**<br><br>(e.g., reach barriers/ enablers improving access/ cost implications influencing delivery)	**Behavior outcomes**<br><br>(e.g., adherence issues based on cultural practices, late presentation due to technologies available like 10-dose vials only)	**Individual outcomes**<br><br>(e.g., lowered risk of MTCT hepatitis B of investigated sample)	**Population level-outcomes**<br><br>(e.g., evidence better health seeking behaviors among pregnant women, decreased hepatitis B incidence of under 5 in the country)	**Non-health outcomes**<br><br>(e.g., less financial expense on chronic hepatitis sufferers, improved, health promotion among pregnant women and mothers)
