# Supplementary material for: Towards contextualized complex systems approaches to scaling-up hepatitis B birth-dose vaccination in the African region: a qualitative systematic review
Source: Front Public Health. 2024 Oct 24;12:1389633. doi: 10.3389/fpubh.2024.1389633 (PMC11540787; doi:10.3389/fpubh.2024.1389633)
Supplement: Supplementary file 4 [file Data_Sheet_4.docx]

**Supplementary File 4**. Characteristics of included studies

| Study No. | Author | Year | Title | Country | Study design | National policy on hepatitis B birth-dose vaccination* | Vaccination implementation strategy in study: Universal *vs* selective | Population group | Description of population | Study sample | Primary objective of the study |
| --- | --- | --- | --- | --- | --- | --- | --- | --- | --- | --- | --- |
| 1 | Accrombessi et al | 2019 | Assessment of the anti-HBs antibody response in Beninese infants following 4 doses of HBV vaccine, including administration at birth, compared to the standard 3 doses regime: a cross-sectional survey | Benin | Cross-sectional | No | Universal | Mother-infant pairs | 9-month-old infant and mother pairs | 140 pairs | **Vaccine efficacy** of hepatitis B birth-dose included regiment |
| 2 | Aina et al | 2017 | Preliminary results from direct-to-facility vaccine deliveries in Kano, Nigeria | Nigeria | Mixed methods (retrospective review) | Yes | Universal | Health facilities | Sentinel health facilities involved in vaccination services | 27 | **Evaluation** of vaccine supply program |
| 3 | Allison et al | 2017 | Hepatitis B vaccine birth dose coverage correlates worldwide with rates of institutional deliveries and skilled attendance at birth | Global | Ecological study | Yes | Universal | Countries | Countries providing universal hepatitis B birth-dose vaccination | 83 | **Factors** associated with hepatitis B birth-dose implementation |
| 4 | Bagney et al | 2015 | Evaluation of midwives' practices for the prevention of mother-to-child transmission of hepatitis B in Abidjan (Ivory Coast) | Ivory Cost | Cross-sectional | No | Selective | Midwives | Midwives recruited from different levels of health facilities | 197 | **Knowledge, attitude and practice** of HCWs regarding PMTCT of hepatitis B practices |
| 5 | Bassoum et al | 2021 | Factors associated with co-administration of birth dose vaccines in Senegalese children | Senegal | Cross-sectional | Yes | Universal | Children | Children aged 12-23 months | 726 | **Factors** associated with implementation of birth-dose vaccines |
| 6 | Bassoum et al | 2022 | Vaccination against tuberculosis, polio and hepatitis B at birth in Podor health district, Northern Senegal: cross-sectional study of vaccination coverage and its associated factors | Senegal | Cross-sectional | Yes | Universal | Children | Children aged 12-23 months | 832 | **Vaccination coverage** and the **factors** associated with **timely** implementation |
| 7 | Chang et al | 2019 | Survey of Impediments to Prevention of Mother-to-infant Transmission of Hepatitis B Virus by International Societies | Global | Mixed methods | Unspecified | Unreported | Countries | Local paediatric HBV experts representing country/ region, and global members of the societies of FISPGHAN | 63  (13 African) | Identifying **obstacles** to hepatitis b vaccination implementation |
| 8 | Dagnew et al | 2020 | Knowledge, attitude and associated factors towards vertical transmission of hepatitis B virus among pregnant women attending antenatal care in tertiary hospitals in Amhara region, northwest Ethiopia: A cross-sectional study | Ethiopia | Cross-sectional survey | No | Unreported | Pregnant women | Pregnant women attending ANC | 1121 | **Knowledge, attitude and practice** of end users regarding PMTCT of hepatitis B practices |
| 9 | Djaogol et al | 2019 | Prevention and care of hepatitis B in the rural region of Fatick in Senegal: a healthcare workers’ perspective using a mixed methods approach | Senegal | Mixed methods survey | Yes | Universal | HCWs | HCWs involved in activities related to maternal and child health | 98 | **Knowledge, attitude and practice** of HCWs regarding implementing hepatitis B PMTCT activities |
| 10 | Goodman et al | 2013 | Health workers sensitization: effects on perceived quality of immunization services among mothers of under five children in Ilorin, North Central Nigeria | Nigeria | Quasi-experimental | Yes | Universal | Mothers | Mothers of children under 5 years old attending immunization clinic for the vaccines of HBV/OPV/BCG and DPT/OPV/BCG | 300 | **Perceived quality** of immunization services of mothers after HCW sensitization |
| 11 | Guingane et al | 2020 | Identifying gaps across the cascade of care for the prevention of HBV mother-to-child transmission in Burkina Faso: Findings from the real world | Burkina Faso | Prospective cohort | No | Selective | Pregnant women | Pregnant women attending ante-natal consultation at one of the 9 first-level health centers in the health district | 2220 | **Evaluation** of PMTCT hepatitis B program in real life setting |
| 12 | Hagan et al | 2019 | Selective Hepatitis B Birth-Dose Vaccination in São Tomé and Príncipe: A Program Assessment and Cost-Effectiveness Study | São Tomé and Príncipe | Qualitative: Field assessment | Yes | Selective | Health facilities | Health facilities providing maternity services, including key informants in MoH, representatives of partners and representative organizations. | 5 | **Evaluation** of selective hepatitis B birth-dose vaccination program |
| 13 | Jaquet et al | 2017 | Prevention and Care of Hepatitis B in Senegal: Awareness and Attitudes of Medical Practitioners | Senegal | Cross-sectional survey | No | Universal | Medical doctors | Certified medical doctors (specialists and general practitioners) practicing in the public sector of 3 major urban areas | 127 | **Knowledge, attitude and practice** of HCWs on HBV infection |
| 14 | Loarec et al | 2022 | Prevention of mother-to-child transmission of hepatitis B virus in antenatal care and maternity services, Mozambique | Mozambique | Retrospective cohort | No | Selective | Pregnant women | Pregnant women (and their new-born infants) attending Chamanculo General Hospital | 6775 | **Evaluation** of PMTCT hepatitis B program in real life setting |
| 15 | Miyahara et al | 2016 | Barriers to timely administration of birth dose vaccines in The Gambia, West Africa | The Gambia | Prospective cohort | Yes | Universal | Infants | Infants born in the Farafenni Health and Demographic Surveillance System between 2004-2014 | 10851 | **Coverage, timeliness and factors** associated with birth-dose vaccine implementation |
| 16 | Nankya-Mutyoba et al | 2021 | Hepatitis B birth dose vaccination for newborns in Uganda: A qualitative inquiry on pregnant women’s perceptions, barriers and preferences | Uganda | Qualitative | No | Unreported | Pregnant women | Pregnant women attending routine ante-natal clinic visits in Central Uganda | 70 | **Awareness, barriers, perceptions and preference** of pregnant women regarding hepatitis B birth-dose vaccination |
| 17 | Okenwa et al | 2019 | Maternal reasons for non-receipt of valid Hepatitis B birth dose among mother-infant pairs attending routine immunization clinics, South-east, Nigeria | Nigeria | Cross-sectional survey | Yes | Universal | Mother-infant pairs | Mother-infant pairs attending immunization clinics in Enugu State of Nigeria | 344 | **Barriers** to birth-dose implementation as identified by mothers |
| 18 | Okenwa et al | 2020 | Maternal knowledge and infant uptake of valid hepatitis B vaccine birth dose at routine immunization clinics in Enugu State – Nigeria | Nigeria | Cross-sectional survey | Yes | Universal | Mother-infant pairs | Mother-infant pairs attending immunization clinics in Enugu State of Nigeria | 366 | **Knowledge, awareness** and **factors** associated with uptake of **timely** hepatitis B birth-dose vaccination |
| 19 | Olakunde et al | 2021 | The coverage of hepatitis B birth dose vaccination in Nigeria: Does the place of delivery matter? | Nigeria | Cross-sectional secondary analysis | Yes | Universal | Children | Children aged 12 – 23 months | 6143 | Vaccine **coverage** and the **associated** influence of place of delivery |
| 20 | Périères et al | 2021 | Hepatitis B Vaccination in Senegalese Children: Coverage, Timeliness, and Sociodemographic Determinants of Non-Adherence to Immunisation Schedules (ANRS 12356 AmBASS Survey) | Senegal | Cross-sectional survey (part of the ANRS 12356 AmBASS survey) | Yes | Universal | Children | Children from the AmBASS survey born on or after 1 January 2016 (year of national hepatitis B birth-dose implementation) | 241 | **Coverage** and associated **factors** related to non-adherence to **timely** vaccination. |
| 21 | Sadoh et al | 2014 | Hepatitis B infection among Nigerian children admitted to a children’s emergency room | Nigeria | Cross-sectional descriptive study | Yes | Universal | Children | All children aged between 2 months and 15 years admitted to children’s emergency room of the University of Benin Teaching Hospital | 150 | **HBV Prevalence,** and vaccination coverage **and timeliness** |
| 22 | Ibrahim et al | 2022 | Factors associated with timeliness of hepatitis B birth dose: A cross-sectional study in North-Western Nigeria | Nigeria | Cross-sectional | Yes | Universal | Mother-infant pairs | Infants and their respective mothers/caregivers presenting at the immunization clinic for their first vaccination dose | 400 | **Factors** associated with **timely** vaccine implementation |
| 23 | Ibraheem et al | 2022 | Assessment of the Timely Administration of Birth Dose Vaccines in Northern Nigeria and Associated Factors | Nigeria | Cross-sectional study (secondary analysis) | Yes | Universal | Mother-infant pairs | Mother/caregiver and infant pair presenting for birth-dose vaccines at the 5 different immunization centers in 5 different Northern Nigerian states | 1952 | **Factors** associated with **timely** vaccine implementation |
| 24 | Ibraheem et al | 2019 | Determinants of Timely Presentation for Birth Dose Vaccination at an Immunization Centre in North-central Nigeria | Nigeria | Cross-sectional | Yes | Universal | Mother-infant pairs | Mother-infant pairs attending immunization centre of General Hospital, Ilorin for first vaccination. | 480 | **Factors** associated with **timely** vaccine implementation |
| 25 | Sadoh et al | 2013 | Factors contributing to delay in commencement of immunisation in Nigerian infants | Nigeria | Cross-sectional descriptive study | Yes | Universal | Mother-infant pairs | Mother-infant pairs presenting for the commencement of immunization at the Child Welfare clinic of the Institute of Child Health, University of Benin, Benin City. | 153 | **Factors and maternal reasons** associated with vaccine implementation, specifically **timeliness** |
| 26 | Danjuma et al | 2020 | At-Birth Vaccination Timeliness: An Analysis of Inborns in the Highlands of Jos, North-Central Nigeria | Nigeria | Cross-sectional | Yes | Universal | Mother-infant pairs | Mother/guardian- new-born (birth weight ≥ 1.8kg) pair in the lay-wards of Jos University Teaching Hospital and Fertile Ground Hospital | 355 | **Factors and obstacles** associated with **timely** vaccine implementation |
| 27 | Bada et al | 2022 | Factors associated with receipt of a timely infant birth dose of hepatitis B vaccine at a tertiary hospital in North-Central Nigeria | Nigeria | Retrospective cohort (secondary analysis) | Yes | Universal | Mother-infant pairs | Mother-infant pairs at a tertiary hospital in North-Central Nigeria | 409 | **Factors** associated with **timely** hepatitis B birth-dose vaccine implementation |
| 28 | Moturi et al | 2018 | Implementing a birth dose of hepatitis B vaccine in Africa: Findings from assessments in 5 countries | Nigeria, Namibia, Botswana, The Gambia, São Tomé and Príncipe | Cross-sectional | Yes | Universal (Nigeria, Namibia, Botswana, The Gambia) and Selective (São Tomé and Príncipe) | Health Facilities | Health facilities providing delivery services | 78 | **Knowledge, attitudes,** and **practices** among HCWs surrounding hepatitis B birth-dose vaccination |
| **Hepatitis B birth-dose vaccination availability at the time the studies were conducted. HCWs- health care workers; PMTCT- prevention of mother to child transmission; MoH- Ministry of Health; AmBASS survey- this is a large population-based survey which aims to study the epidemiology, and socioeconomic and public health impacts of HBV chronic infection in specific contexts; FISPGHAN- Federation of the international societies for pediatric gastroenterology, hepatology and nutrition; (DPT)/HBV/OPV/BCG- (Diphtheria, pertussis, tetanus)/hepatitis B virus / oral polio virus/tuberculosis* | | | | | | | | | | | |
